# Genetic control of grain amino acid composition in a UK soft wheat mapping population

**DOI:** 10.1002/tpg2.20335

**Published:** 2023-05-03

**Authors:** Joseph Oddy, Monika Chhetry, Rajani Awal, John Addy, Mark Wilkinson, Dan Smith, Robert King, Chris Hall, Rebecca Testa, Eve Murray, Sarah Raffan, Tanya Y. Curtis, Luzie Wingen, Simon Griffiths, Simon Berry, J. Stephen Elmore, Nicholas Cryer, Isabel Moreira de Almeida, Nigel G. Halford

**Affiliations:** ^1^ Rothamsted Research Harpenden UK; ^2^ John Innes Centre Norwich Research Park Norwich UK; ^3^ Curtis Analytics Limited Sandwich UK; ^4^ Limagrain UK Ltd Market Rasen UK; ^5^ Department of Food & Nutritional Sciences University of Reading Reading UK; ^6^ Mondelēz UK R&D Ltd Bournville UK; ^7^ Mondelēz R&D International Saclay France

## Abstract

Wheat (*Triticum aestivum* L.) is a major source of nutrients for populations across the globe, but the amino acid composition of wheat grain does not provide optimal nutrition. The nutritional value of wheat grain is limited by low concentrations of lysine (the most limiting essential amino acid) and high concentrations of free asparagine (precursor to the processing contaminant acrylamide). There are currently few available solutions for asparagine reduction and lysine biofortification through breeding. In this study, we investigated the genetic architecture controlling grain free amino acid composition and its relationship to other traits in a Robigus × Claire doubled haploid population. Multivariate analysis of amino acids and other traits showed that the two groups are largely independent of one another, with the largest effect on amino acids being from the environment. Linkage analysis of the population allowed identification of quantitative trait loci (QTL) controlling free amino acids and other traits, and this was compared against genomic prediction methods. Following identification of a QTL controlling free lysine content, wheat pangenome resources facilitated analysis of candidate genes in this region of the genome. These findings can be used to select appropriate strategies for lysine biofortification and free asparagine reduction in wheat breeding programs.

## Core Ideas

- High free asparagine and low lysine concentrations limit the nutritional value of wheat grain.
- Investigation of a biparental mapping population formed from the UK soft wheats Claire and Robigus.
- Breeding for lower free asparagine and higher lysine using Claire and Robigus diversity is possible but limited.

**Abbreviations**
GS: genomic selection
HFN: Hagberg falling number
KHI: kernel hardness index
PCA: principal components analysis
QTL: quantitative trait locus

## 1 INTRODUCTION

The nutritional quality of wheat (*Triticum aestivum* L.) has profound impacts on human health. As one of the largest sources of average daily calorie intake in the world (18.2% in 2019; FAO, 2021), wheat is an essential source of macro‐ and micronutrients. In 2019, 19.5% of average daily global protein intake was estimated to be provided by wheat‐based foods (FAO, 2021). Similarly, between 2008 and 2017 in the UK, over 25% of average daily fiber intake was provided by wheat‐based foods (Gressier & Frost, 2022). Wheat flour is often fortified to increase its nutrient content. In the UK, wheat is fortified with calcium, iron, thiamine, niacin, and, most recently, folate (DEFRA, 1998; Department of Health and Social Care UK Government, 2021). The quantities of different macro‐ and micronutrients in wheat can have large impacts on population health because of the scale at which wheat products are consumed. For example, it is estimated that the addition of folate to UK flour will lead to a 20% decrease in neural tube defects in babies (Department of Health and Social Care UK Government, 2021). Consequently, it is essential to ensure that the nutritional profile of wheat is as beneficial as it can be for human health.

One way in which the nutritional profile of wheat can be improved is via optimization of its amino acid composition, with the concentrations of lysine and asparagine as most important. Free (soluble, nonprotein) asparagine can be converted into the processing contaminant, acrylamide, during high‐temperature cooking and processing, and this has led to ongoing efforts to reduce free asparagine concentration (Oddy et al., 2022). Lysine, on the other hand, is not produced endogenously by humans or other monogastric animals, making it an essential amino acid in the diet, but it is present in only small quantities in wheat and other cereal grain and populations reliant on cereals for their nutrition may suffer from lysine deficiency (Galili & Amir, 2013). Indeed, fortifying wheat flour by adding lysine has been shown to improve indices of nutritional status in clinical trials in Pakistan, northern China, and Syria (Hussain et al., 2004; Zhao et al., 2004; Ghosh et al., 2008). Flour fortification is unlikely to be a sustainable solution in developing countries and it would be much cheaper and more efficient to increase the intrinsic lysine content of wheat grain. Therefore, the amino acid composition of wheat grain could be optimized both by decreasing grain free asparagine content and increasing lysine content.

In recent years, studies have investigated genetic strategies for the reduction of free asparagine content in wheat grain. Induced and natural variation in the asparagine synthetase 2 genes, for example, has been found to impact significantly on free asparagine content (Alarcon‐Reverte et al., 2022; Oddy et al., 2021; Raffan et al., 2021) and quantitative trait loci (QTLs) for grain asparagine content have been identified from previous genome‐wide association studies (GWAS) (Emebiri, 2014; Peng et al., 2018; Rapp et al., 2018). However, the small number of stable QTL available to breeders limits the progress that can be made to reduce grain asparagine content in breeding programs and no genetic strategies for soft (biscuit) wheat specifically have been investigated. Similarly, there are limited strategies currently available for increasing lysine content in wheat grain. Lysine biofortification via QTL identification and marker‐assisted breeding has been studied extensively in both rice (*Oryza sativa* L.; Jang et al., 2020; Wang et al., 2008; Yoo, 2017; Zhong et al., 2011) and maize (*Zea mays* L.; Prasanna et al., 2020), but only two studies have previously investigated lysine biofortification in wheat through association studies. Peng et al. (2018) successfully identified QTL controlling free lysine and Jiang et al. (2013) identified QTL for total lysine.

Consequently, the aim of this study was to investigate QTL, genomic prediction (GP) accuracy, and candidate genes controlling the free amino acid composition of wheat grain in a soft wheat mapping population developed from the varieties Claire and Robigus. Like many UK varieties, these parents both lack the B genome homologue of the asparagine synthetase‐2 gene, *TaASN‐B2* (TraesLDM3B03G01566640 in variety Landmark), the presence/absence of which is a known source of grain asparagine content variation (Oddy et al., 2021). This mapping population, therefore, represents a useful resource for identifying additional variation. Claire and Robigus are also represented by scaffold‐level genome assemblies in the wheat pangenome, facilitating candidate gene analysis. Furthermore, we investigated other traits, such as grain size, hardness, and Hagberg falling number (HFN), to determine whether QTL controlling nutritional traits overlapped with those controlling other traits of interest.

**Core Ideas**

- High free asparagine and low lysine concentrations limit the nutritional value of wheat grain.
- Investigation of a biparental mapping population formed from the UK soft wheats Claire and Robigus.
- Breeding for lower free asparagine and higher lysine using Claire and Robigus diversity is possible but limited.

## 2 MATERIALS AND METHODS

### 2.1 **Production of** doubled haploid **lines**

Doubled haploid lines (171) of Robigus × Claire were produced using a modified Knox et al. (2000) method. Wheat spikes were emasculated between growth stages GS55 and GS59. Once the stigma was receptive it was fertilized with freshly shed donor maize pollen. After 1 day, wheat florets were treated with Dicamba (20mgL^−1^) (Sigma‐Aldrich, D5417) and injected into the plant stem (100 mg L^−1^). Developing embryos were excised between 14 and 21 days. Under aseptic conditions, seeds were removed from the spikelets, surface sterilized with 70% (v/v) ethanol (EtOH) for 1 min, rinsed with sterile distilled water, and immersed in 20% (v/v) commercial bleach solution with a few drops of Tween 20 for 20 min. They were then rinsed with sterile distilled water three times.

Haploid embryos were excised and grown on 90 mm Petri dishes in the dark on Gamborg's B5 media with minimal organics (Gamborg et al., 1968), 2% (w/v) sucrose, pH 5.8, 9 g L^−1^ Difco bactoagar at 20°C. When showing signs of germination, embryos were transferred to a light incubator at 20°C. Any nongerminated 1‐month‐old embryos were given cold shock treatment at 4°C for 7 days to promote germination. Germinated plantlets were vernalized for 4 weeks and were grown in the glasshouse until the 4‐tiller stage. Plants were then given colchicine (C9754; Sigma‐Aldrich) treatment for 5–6 h in the light at room temperature, washed and transplanted to soil, acclimatized, and grown in a glasshouse. The mapping population was genotyped by Limagrain using a proprietary single‐nucleotide polymorphism (SNP) array. The genetic map comprising 872 loci was constructed using MSTMap Online (http://mstmap.org/).

The mapping population was grown in field trials at the John Innes Centre Morley Mill Hill field site (52°33′15.1″N 1°01′59.2″E) in 2017–2018 (abbreviated as H18) and at the Church Farm field site (52°38′N 1°10′E) in 2018–2019 (abbreviated as H19). All 171 Doubled Haploid lines of the mapping population were grown in each trial. Within each trial, one replicate of each line was drilled in 6 m^2^ plots in a completely randomized design. The H18 field trial was drilled on September 21, 2017 and harvested on August 1, 2018. The H19 field trial was drilled on the September 14, 2018 and harvested on the August 12, 2019. Growth habit, heading date, plant height, and yield traits were scored in the field.

DoubleTop fertilizer (27N 30SO_3_) was applied at a rate of 150 kg/ha on the March 20, 2018 for H18 and the February 23, 2019 for H19. In both trials, slug control pellets were applied at a rate of 7 kg/ha after drilling to control slug pests (Gusto (metaldehyde) pellets on September 27, 2017 for H18 and Sluxx (Ferric phosphate) pellets on September 24, 2018 for H19). Herbicide mixtures were applied in autumn (November 21, 2017 for H18 and September 24, 2018 for H19) and spring (May 23, 2018 for H18 and March 19, 2019 for H19) for both trials to control weeds.

### 2.2 Phenotyping

Grain diameter, kernel hardness index (KHI), and grain weight measurements were recorded for 300 kernels from each line in the population using a Perten Single Kernel Classification System (SKCS) 4100 (Calibre Control International Ltd.). Grain length (mm), width (mm), and area (mm^2^) measurements were recorded in triplicate for each sample using a MARVIN Seed Analyser and software Marvin 4.0 (MARViTECH GmbH). Grain samples were milled to wholemeal flour in a coffee grinder and flour moisture content was recorded using a Minispec nuclear magnetic resonance (NMR) analyzer (Minispec Mq10, Bruker Inc.). Hagberg falling number measurements were recorded using an FN 1000 as the average of two technical replicates (Perten), adjusting for flour moisture content as required according to manufacturer's instructions. Amino acid analysis was performed on wholemeal flour samples by HPLC as described previously (Raffan et al., 2021) by Curtis Analytics. Briefly, free amino acids were extracted from 0.5 g of wholemeal flour and underwent precolumn derivatization (Curtis et al., 2018). Samples were then run on an HPLC system identically to previously described (Raffan et al., 2021). Three technical replicates were taken for each sample for amino acid measurement.

### 2.3 Phenotypic data analysis

Skewness and kurtosis were measured for all variables in each environment and normal plots visually inspected in Genstat (VSN International, 2021) to determine if variables required transformation. The data were appropriately transformed according to their distribution if necessary (see Tables S1 and S2 for details of transformations). Subsequent analyses were performed on transformed variables unless otherwise stated. Plotting was performed in R (R Core Team, 2021) with the packages ggplot2 (Wickham, 2016), tidyverse (Wickham et al., 2019), and cowplot (Wilke, 2020).

Broad‐sense heritability for each trait was estimated as described in Covarrubias‐Pazaran (2019) using the packages dplyr (Wickham et al., 2022) and lme4 (Bates et al., 2015). Kendall rank correlation coefficients were performed on nontransformed data and adjusted *p‐*values (Bonferroni correction) were calculated for plotting using R (R Core Team, 2021) and the package corrplot (Wei & Simko, 2021). Principal component analysis was performed on untransformed, scaled variables using the package factoextra (Kassambara & Mundt, 2020). Correlation network analysis was performed and plotted by filtering for significant correlations where *p* < 0.001 using Kendall correlation with Bonferroni correction using the packages corrr (Kuhn et al., 2020), igraph (Csardi & Nepusz, 2006), and ggraph (Pedersen, 2021).

Bayesian modeling was performed on untransformed variables in R using the package rstanarm (Goodrich et al., 2020). Variables were scaled before modeling and individual linear models for each predictor variable were created to guide the selection of informative priors. Simulations of the posterior distribution were subsequently performed to check model fit and intervals were plotted using the package bayesplot (Gabry & Mahr, 2022). *R* ^2^ estimates were obtained by taking the median of leave‐one‐out cross validation adjusted estimates.

### 2.4 Linkage analysis

Single‐environment linkage analysis was performed in R using packages qtl (Broman et al., 2003) and qtl2 (Broman et al., 2019). Single‐environment linkage analysis was made into an interactive app using the packages shiny (Chang et al., 2021), plyr (Wickham, 2011), and rsconnect (Atkins et al., 2021) (accessible at https://t9onwp‐wheatworker.shinyapps.io/QTL_Browser/ and in Supporting Information 1). As before, simple interval mapping (SIM) was performed first to identify covariates for use in composite interval mapping (CIM). Identified QTL from CIM were then used to create single QTL models as well as additive QTL models. Upper and lower 95% confidence intervals for QTL location were calculated using the Bayesian credible interval method in R/qtl and expanded to the closest markers. Pseudomarkers were generated every 2 cM in the map and the minimum marker covariate proximity was set at 20 cM. A logarithm of the odds (LOD) score of 3 was used as the significance threshold.

Multi‐environment single trait linkage analysis was performed in Genstat for each trait to detect QTL present in both environments, following selection of the most appropriate variance–covariance model according to the Bayesian information criterion. SIM was initially performed to identify putative QTL. These QTLs were then used as covariates in CIM. QTL identified from CIM was then used to construct the final QTL models. Pseudomarkers were generated every 2 cM in the map. The minimum cofactor proximity was set at 30 cM, and the minimum separation for selected QTL was set at 20 cM. Significance thresholds were determined by the Li and Ji's (2005) method with a genome‐wide significance level of 0.05.

### 2.5 Genomic prediction

GP was performed for each trait via fivefold cross validation with 10,000 permutations using the R package rrBLUP (Endelman, 2011). The “mixed.solve” function within this package was used to estimate marker effects for each trait, with the identity matrix being left unspecified. Pearson correlation coefficients were calculated for the results from the training and testing datasets to estimate GP accuracy. For within year prediction estimates, training and testing datasets came from the same trial. For between year prediction estimates, training and testing datasets were from different trials. Further detail is available as R markdown in Supporting Information 2. Scripts were submitted to the high‐performance computing cluster at Rothamsted Research via SLURM for execution.

### 2.6 Candidate gene analysis

The gene content of the lysine QTL was determined for all wheat pangenome varieties at chromosome scale assembly by identifying the location of the markers in these varieties and extracting genes from Ensembl Biomart (Howe et al., 2021). Genes residing within the region in variety Chinese Spring v1.0 were submitted to KnetMiner (https://knetminer.com/Triticum_aestivum/) (Hassani‐Pak et al., 2021) for ranking on relevant keywords (“Lysine,” “Storage proteins”). Expression of the top hits was then investigated in expVIP (Borrill et al., 2016) to further narrow down plausible candidate genes. Transcript per million (TPM) data for the Azhurnaya developmental time‐course experiment were extracted from expVIP for plotting in R using the package pheatmap (Kolde, 2019). Corresponding Claire and Robigus genes were then identified from these Chinese Spring candidate genes in Ensembl and pairwise aligned via BLAST using Geneious Prime 2020.1.2 to identify variation.

## 3 RESULTS

### 3.1 Phenotypic analysis

**FIGURE 1:**
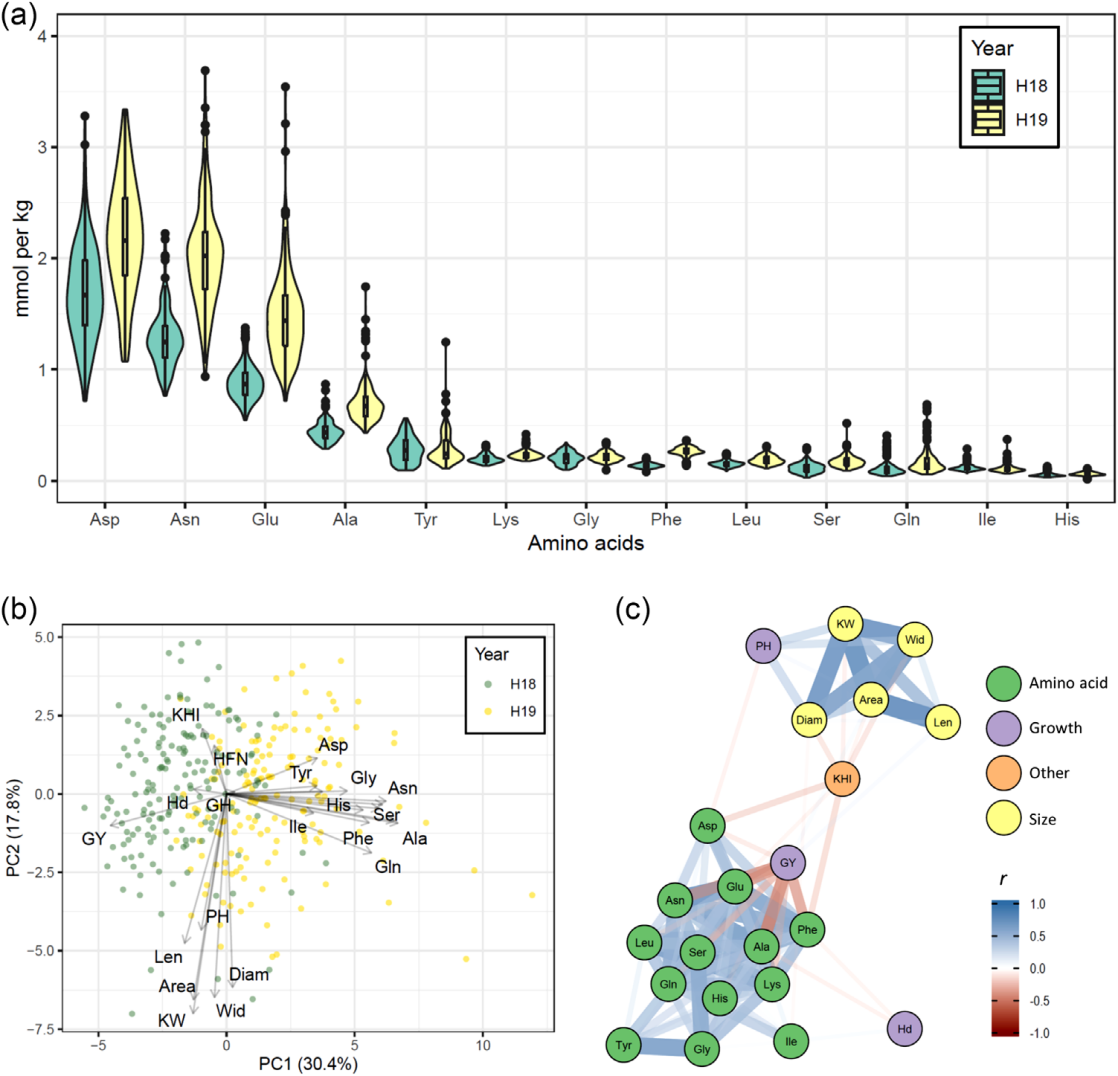
Characterization of the Robigus × Claire mapping population. (a) Measurements of amino acids in the 2017–2018 (H18) and 2018–2019 (H19) harvest years. (b) Principal component analysis of all traits in both years along the first two principal components. (c) Correlation network analysis of all traits across both years (GH omitted, Kendall correlation, only links with significance <0.001 shown). GH (growth habit), GY (grain yield), Hd (heading date), HFN (Hagberg Falling Number), KHI (kernel hardness index), KW (kernel weight), PH (plant height).

**FIGURE 2:**
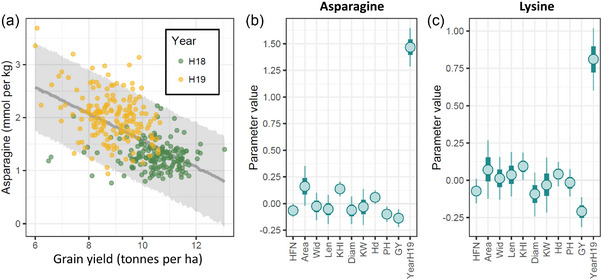
Relationships between free asparagine/lysine and other agronomic measurements. (a) Linear modeling of free asparagine content against grain yield. The gray shaded ribbon shows 95% prediction intervals sampled from the posterior distribution. (b and c) Parameter values from multiple linear modeling of asparagine (b) and lysine (c) as explained by other traits measured in this population. HFN, Hagberg falling number; KHI, kernel hardness index.

We measured free amino acid concentrations and other traits of interest in the Robigus × Claire mapping population from field trials grown in 2017–2018 (H18) and 2018–2019 (H19) (Figure 1; Figure S1). Aspartic acid, asparagine, and glutamic acid were the most abundant of the free amino acids measured, with concentrations of free amino acids consistently higher in H19 than in H18 (Figure 1a). Principal component analysis (PCA) revealed harvest year to be a key driver of variation in this dataset (Figure 1b) and, notably, the second harvest year (H19) also showed lower yield alongside the increased free amino acid content of the grain (Figure 1b). PCA and correlation network analysis revealed that most of the other traits measured here were uncorrelated with the amino acids (Figure 1b,c; Figures S2 andS3), except for grain yield which showed negative correlations with a subset of amino acids (Figure 1b,c; Figure 2a).

To understand whether any of the traits we measured could predict free asparagine or lysine content in the grain, we constructed Bayesian linear models with the traits and harvest year as explanatory variables (Figure 2b,c). In both the free asparagine (Figure 2b) and lysine (Figure 2c) models, environment had the greatest effect, whereas other variables had little explanatory power. Nevertheless, the variance explained in the models was still reasonable for asparagine at 56.5%, but only 22.2% for lysine.

### 3.2 QTL analysis

Broad‐sense heritability estimates varied substantially between the different amino acids, with free asparagine and lysine showing heritability estimates of 0.60 and 0.45, respectively (Table S1). Aspartic acid showed the highest heritability of the amino acids measured here, with an estimate of 0.82. Heritability estimates for the size traits were generally very high, as expected, and correlation of these values between years was also stronger than the correlation of amino acids between years (Table S1).

**FIGURE 3:**
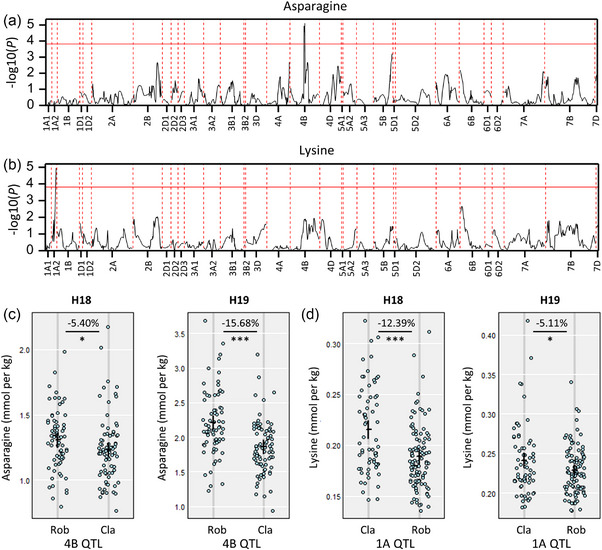
Identification of quantitative trait locus (QTL) controlling free asparagine and free lysine. (a) Multi‐environment genome scan plot for asparagine. (b) Multi‐environment genome scan plot for lysine. (c) Impact of the asparagine QTL on free asparagine concentrations in both field trials. (d) Impact of the lysine QTL on free lysine concentrations in both field trials. Error bars show plus and minus two times standard error of the mean. Significance values are taken from the corresponding years of the multi‐environment linkage analysis.

We identified QTL for grain free asparagine content and lysine content on chromosomes 4B and 1A, respectively (Figure 3a,b; Table 1), which had significant effects across both environments but were also affected by QTL by environment effects (Figure 3c; Figure 3d; Table 1; Table S2). The asparagine QTL on 4B explained 2.6% of the variance in H18, when free asparagine concentrations were lower overall, whereas it explained 14.8% of the variance in H19, when free asparagine concentrations were elevated (Table 1). In both years, the Robigus allele was associated with the higher free asparagine concentrations. In contrast, the lysine QTL on 1A explained 12.1% of the variance in H18, when free lysine was lower overall, and only 2.6% of the variance in H19, when free lysine concentrations were elevated. The Claire allele was associated with higher free lysine concentrations in both years in this case. Multi‐environment linkage analysis of amino acid and grain measurements revealed many QTL controlling the other amino acids and traits as well (Table 1; Tables S2 and S3).

**TABLE 1:** Multi‐environment quantitative trait locus (QTL) for measured amino acids.

**Trait**	**Multi‐environment single trait linkage analysis (H18 and H19)**							
	**Marker**	**Chr**.	**cM**	**Mbp**	**−log_10_(*p*)**	**H18 (%)**	**H19 (%)**	**High val**.
Ala	WC.0223839	7B	211.2	719	5.03	7.1	5.7	Robigus
Asn	WC.0221262	4B	114.47	601	5.96	2.6	14.8	Robigus
Asp	WC.0218489	1B	54.4	530	5.4	8	5.9	Claire
	WC.0214359	3A2	2.3	738	7.95	7.3	15.3	Robigus
	WC.0221037	4A	148.8	703	8.08	12.6	9.3	Claire
	WC.0227146	4D	48.8	16	3.7	5.5	4.1	Claire
Gln	WC.0221302	4B	103.7	547	3.5	5.4	4.5	Robigus
	WC.0228471	6B	19.7	25	5.09	8.2	6.7	Claire
Glu	WC.0221329	4B	100.8	518	4.27	3.7	10.1	Robigus
Gly	WC.0226796	4B	155.2	327	4.26	3.2	5.3	Robigus
Iso	WC.0223785	7B	211.2	717	3.6	6.8	3.7	Robigus
Lys	WC.0218011	1A2	27.3	593	4.95	12.1	2.6	Claire
Phe	WC.0220622	3B1	78.1	116	3.83	6.2	5.6	Robigus

Abbreviations: Chr., chromosome; cM, centimorgan; H18/H19 (%), percentage of variation explained by QTL in respective year; High val., parental line carrying the allele responsible for the higher value; Mbp, megabase pair location in Chinese Spring v1.0.

The QTL controlling asparagine on chromosome 4B appeared to overlap with QTL for glutamine, glutamic acid, and glycine (Table 1). Each of these QTL had a greater effect in H18 than in H19 and the Robigus allele was associated with the higher value in each case as well, suggesting that these QTL are caused by the same variant. These QTL were also located near to a QTL for KHI and more distantly to QTL for grain diameter, plant height, and grain weight (Table 2, Figure S4), which are likely caused by the *Rht‐B1* polymorphism. QTL for aspartic acid also appeared to overlap with QTL for other traits (Table 2). For aspartic acid on 4A and 4D, there are co‐locating HFN QTL, suggesting that these two traits are under the control of the same locus (Figure S5). The location of the QTL on 4D matches the *Rht‐D1* polymorphism between Claire and Robigus found at 18.78 Mbp in Chinese Spring. Of all the amino acids measured in this study, we identified the most QTL controlling aspartic acid (Table 1). Other potential sources of variation underpinning the QTL in this study are presented in Table S4.

**TABLE 2:** Multi‐environment quantitative trait locus (QTL) impacting both amino acids and other traits on chromosomes 4A, 4B, and 4D.

**Chr**.	**Multi‐environment single trait linkage analysis (H18 and H19)**							
	**Trait**	**Marker**	**cM**	**Mbp**	**−log_10_(*p*)**	**H18 (%)**	**H19 (%)**	**High val**.
4A	Asp	WC.0221037	148.8	703	8.08	12.6	9.3	Claire
	KHI	WC.0221037	148.8	703	8.26	14.8	14.7	Robigus
	HFN	WC.0188904	147.1	733	8.24	11.5	10.3	Robigus
	Area	WC.0220938	149.7	709	2.22	3	3.2	Claire
	Length	WC.0221119	149.7	702	7.12	1.8	6.5	Claire
4B	Asn	WC.0221262	114.47	601	5.96	2.6	14.8	Robigus
	KHI	WC.0226741	110.8	594	4.30	4.2	8.6	Robigus
	Diam	WC.0226868	82.5	32	18.53	17.7	18.7	Claire
	Height	WC.0226868	82.5	32	17.98	19.8	20.7	Claire
	Weight	WC.0226868	82.5	32	9.25	12.2	14.1	Claire
4D	Asp	WC.0227146	48.8	16	3.7	5.5	4.1	Claire
	Width	WC.0227146	48.8	16	5.98	8	8.9	Robigus
	Diam	WC.0227146	48.8	16	7.84	7.6	8.1	Robigus
	HFN	WC.0227149	56.9	17	10.92	23.5	5.6	Robigus
	Height	WC.0213051	56.9	17	28.97	27.8	38.9	Robigus

Abbreviations: Chr., chromosome; cM, centimorgan; H18/H19 (%), percentage of variation explained by QTL in respective year; High val., parental line carrying the allele responsible for the higher value; HFN, Hagberg falling number; KHI, kernel hardness index; Mbp, megabase pair location in Chinese Spring v1.0.

### 3.3 Genomic prediction

**FIGURE 4:**
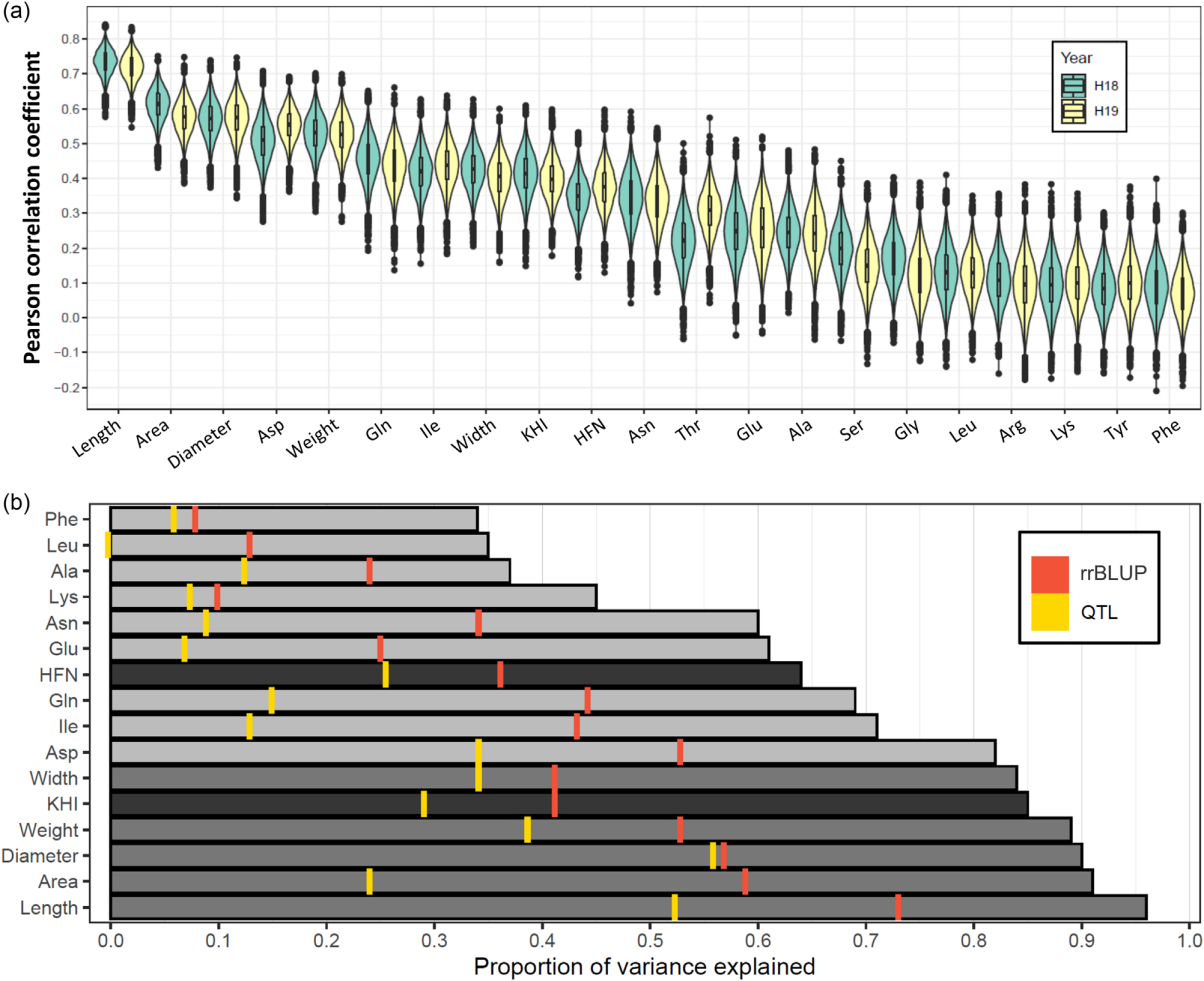
Variation explained by heritability, genomic prediction, and quantitative trait locus (QTL). (a) Genomic prediction accuracy between years. (b) Additive QTL effects and genomic prediction (rrBLUP) accuracy (yellow and red marks, respectively) plotted alongside broad‐sense heritability (shown as bars). Bars are shaded according to the trait group that they belong to (amino acid, size, or other). HFN, Hagberg falling number; KHI, kernel hardness index.

Following our modeling of asparagine and lysine using agronomic measurements and QTL models, we calculated the accuracy of GP for within and between year prediction of traits (Figure 4a; Figure S6; Table S1). Prediction accuracy was more consistent when performed across years rather than within years (Figure S6), so these were used for further interpretation. Prediction accuracy for lysine was the lowest of all traits at a mean accuracy of 0.10, whereas accuracy for asparagine was around 0.34. Of all amino acids, aspartic acid had the greatest prediction accuracy results. Prediction accuracies for the other functional traits were generally higher than the accuracies for amino acids, as expected from the higher heritability of these traits. Comparing the amount of variation explained by GP methods and additive QTL models, we can see that the GP models explain more variance than the additive QTL models for all traits (Figure 4b).

### 3.4 Lysine QTL candidate gene analysis

The gene content and QTL size of the lysine QTL on 1A, the HFN/aspartic acid/KHI QTL on 4A, and the asparagine QTL on 4B differed substantially (Table S5). Due to the size of the 4A and 4B QTL, we were unable to plausibly narrow down candidate genes, whereas the lysine QTL on 1A was much smaller so amenable to further analysis. We investigated the gene content of the lysine QTL for all genomes assembled to chromosome scale in the wheat pangenome and gene content varied to a small extent between the different varieties (Table S6). Most notably, the QTL did not match any locations in variety Julius and matched to an unanchored scaffold in Stanley.

**FIGURE 5:**
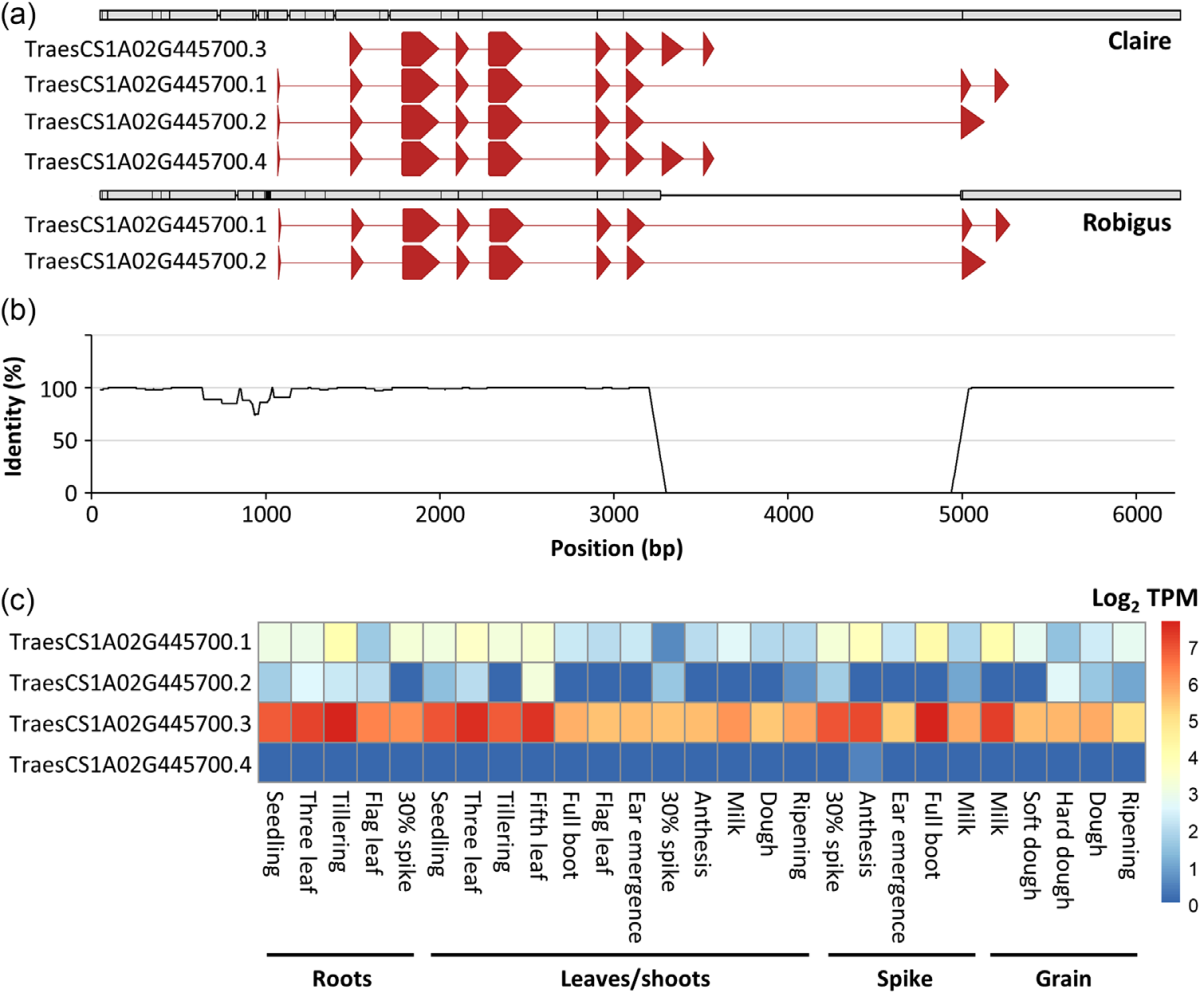
Analysis of the *TaHDT‐A1* candidate gene for lysine quantitative trait locus (QTL) between parents Claire and Robigus. (a) Pairwise alignment of the two genes. (b) Percentage identity calculated as a sliding window average of 100 bp. (c) Expression of the four transcripts throughout development in variety Azhurnaya. TPM, transcript per million.

KnetMiner analysis of the genes residing in Chinese Spring in the lysine QTL was undertaken with relevant keywords to highlight possible candidate genes, and these genes were subsequently investigated for their expression patterns from expVIP. Pairwise analysis of the top KnetMiner hits in the lysine QTL showed that the top hit (TRAESCS1A02G445700) differed between Claire and Robigus. TRAESCS1A02G445700, or *TaHDT‐A1*, has been identified as a member of the histone deacetylase family in wheat. A deletion within the coding sequence of the gene in Robigus means that the most highly expressed transcript cannot be expressed (Figure 5) and the two missing exons from this most highly expressed transcript form a zinc finger/C2H2 DNA binding domain, which is important for transcriptional regulation.

## 4 DISCUSSION

### 4.1 Limited variation in Claire and Robigus for asparagine and lysine improvement

Soft wheat breeding in the UK has relied heavily upon Claire and Robigus as parents since their development in 1999 and 2005, respectively. A recent study found that UK winter wheats developed between 2002 and 2017 could be clustered into four distinct populations, and two of these populations were characterized by their Claire or Robigus heritage (Shorinola et al., 2022). The varieties within these population groups characterized by Claire and Robigus heritage are also almost entirely soft wheat varieties, further emphasizing the importance of these two varieties in UK soft wheat breeding. This large contribution of Claire and Robigus as parents to soft wheat breeding means that opportunities for nutritional improvement have often been limited to variation between these two parents.

Our analysis found that there is variation between Claire and Robigus and that this does impact asparagine and lysine content to a small extent. Asparagine had a moderate heritability (0.60) across both field trials in the study, whereas the heritability for lysine was lower (0.45). One QTL was found for asparagine and lysine each, both explaining less than 10% of the variance on average. The asparagine QTL identified here (peak at 601.4 Mbp in Chinese Spring) lies around 60 Mbp from another QTL (peak at 660.7 Mbp in Chinese Spring) identified by Peng et al. (2018), suggesting that these may coincide, whereas the lysine QTL does not overlap with previously identified QTL. Genomic selection had a predictive ability of 0.34 on average for asparagine, indicating that this method may be better suited for breeding because of the genetic architecture of this trait (many small‐effect QTL). Rapp et al. (2018) also found that genomic selection (GS) had a predictive ability of around 0.5 on average for asparagine, the higher estimate in this study likely due to within environment prediction and analysis of a more diverse mapping population. GS only achieved a predictive ability of 0.10 for lysine, indicating that only incremental advances in lysine content are possible using Claire and Robigus. Previous GWAS studies using more diverse panels have found more, larger effect QTL controlling asparagine and lysine content (Peng et al., 2018; Rapp et al., 2018), indicating that there may be beneficial alleles in more diverse germplasm. Consequently, UK soft wheat germplasm will require diversity beyond Claire and Robigus to make changes to asparagine and lysine content beyond the incremental improvements found here.

### 4.2 Trade‐offs between amino acid content and other traits

Another aspect we wanted to investigate in this population was whether there were any relationships between amino acids and other traits. Amino acids tended to correlate positively with one another and were mostly unrelated to the other measured traits, with the exception of grain yield and kernel hardness index. A negative correlation between grain yield and free asparagine has previously been documented (Xie et al., 2021), but in other experiments the association has been positive (Malunga et al., 2021; Xie et al., 2021). In our analysis, this association mostly arose because of the effect of environment on both yield and asparagine. Environmental stress can lead to decreases in yield while increasing free asparagine, while other variables (e.g., nitrogen fertilizer) can lead to increases in both yield and free asparagine (see Oddy et al. (2022) for review). Our modeling of asparagine through these variables mostly indicated environment as the driving force in our study, but there was still a slight negative association with yield and plant height as well as a slight positive association with kernel hardness. Kernel hardness, like grain free asparagine content, is known to increase with nitrogen application, which may underly this small association with asparagine.

A strong environmental effect on free asparagine concentration has been observed in response to many different stressors (see Oddy et al., 2020 for review) and it is under stressful conditions that the highest asparagine levels are often observed. These increases in grain asparagine concentration vary massively, causing unexpected blips in acrylamide content in food products. These environmentally induced increases pose the greatest threat to food safety and regulatory compliance, so elimination of this environmental response would be of great interest. A weak environmental effect was seen in this study: during the 2018–2019 season, the average amino acid concentrations rose while the yields dropped. Interestingly, the asparagine QTL we identified here had greater effect in this season, enabling reductions of 15.68% in free asparagine concentrations in those lines possessing the Claire allele over those possessing the Robigus allele. This suggests that this QTL may be more effective under more stressful conditions, so selection of the Claire allele at this locus may prove beneficial for reducing the large free asparagine increases observed following environmental stress. This is in contrast to the effect of the *TaASN‐B2* deletion, which has a greater effect when grain asparagine concentrations are lower (Oddy et al., 2021), when plants are not suffering from sulfur deficiency. Future work would therefore benefit from identification of similar QTL that are associated with lowering asparagine content from the high levels seen during stress. This would enable the stacking of alleles that are beneficial under both stress and non‐stress conditions to ensure that free asparagine concentrations are minimized in all environments.

We also wanted to understand whether any QTL controlling amino acid content had pleiotropic effects on other traits. The asparagine QTL we identified on chromosome 4B appeared to overlap with QTL for plant height in the first year, suggesting that there might be an impact of the *Rht‐B1b* allele on asparagine. The *Rht* genes are dwarfing genes used during the green revolution that have many impacts on crop traits beyond height (Casebow et al., 2016) and Claire and Robigus both possess different *Rht* genes on 4B and 4D (Wilkinson et al., 2020). However, this QTL overlap was not present in the second year of analysis when the asparagine QTL had a greater effect, suggesting that the QTL controlling height and asparagine may be distinct. However, a more detailed analysis is required to comprehensively assess the impact of *Rht‐B1* alleles (and dwarfing genes in general) on grain asparagine content. The QTL controlling asparagine did overlap consistently with a QTL for KHI though, with the “increasing allele” belonging to Robigus for both traits. Kernel hardness and free asparagine content are both known to correlate under certain conditions with nitrogen content (Oddy et al., 2022), so this QTL may be linked to nitrogen use efficiency/uptake. The KHI QTL on 4B also exhibited a similar genotype by environment effect pattern to the asparagine QTL, with a greater effect of the QTL observed in the second trial year. Selection for the Claire allele at this QTL would therefore be suitable in the context of soft wheat breeding, where both softer textures and lower asparagine content are desirable.

Interestingly, we found much more genetic control of free aspartic acid concentration in this population compared to the other amino acids. Heritability was high (>0.8), GP accuracy was moderate (>0.5, same as grain weight), and there were four multi‐environment QTL controlling the trait. Two of the QTL controlling aspartic acid also overlapped with QTL controlling HFN. One of these QTL was situated on 4D and overlapped with traits for plant height and grain size as well, indicating that this may be due to *Rht‐D1* allele status, which is known to impact HFN as well as plant height (Fradgley et al., 2022). The second QTL controlling both aspartic acid and HFN was situated on 4A and also overlapped with traits for grain size and KHI. Previous work has identified a major QTL underlying pre‐harvesting sprout (PHS) variation on 4A, but both Claire and Robigus share the same *MKK3‐A* allele which underlies this QTL (Shorinola et al., 2017). Li, Zhang et al. (2021) also identified a PHS QTL in a similar region on 4A but this does not overlap with the region identified here. One possible source of variation underlying the QTL controlling aspartic acid and HFN on 4A is the *Triticum dicoccoides* introgression in Robigus, which matches the region this QTL is found in (Przewieslik‐Allen et al., 2021). The antagonistic relationship between HFN and asparagine at this QTL could be a result of increased HFN reducing proteolysis, and thereby preventing accumulation of free amino acids.

### 4.3 Lysine candidate genes

Scaffold‐level genome assemblies of Claire and Robigus (Walkowiak et al., 2020) enabled us to investigate the lysine QTL in greater depth, identifying the candidate gene *TaHDT‐A1*, encoding a histone deacetylase. The wheat histone deacetylase family is very large, encompassing approximately 50 genes (Jin et al., 2021; Li et al., 2022). Histone deacetylases function mainly to inhibit gene expression because histone deacetylation causes chromatin condensation, with roles in many different developmental processes and environmental responses. In wheat, it is known that differences in grain lysine content can be caused by differential expression of lysine‐poor storage proteins (prolamins). Gill‐Humanes et al. (2014), for example, identified downregulation of gliadins (a class of prolamins) as a method of increasing lysine content in wheat, and Moehs et al. (2019) showed that mutation of wheat prolamin binding factor (WPBF), a DNA‐binding with one finger (DOF)‐class transcription factor, increased lysine concentration. Lower prolamin protein content is also associated with increased lysine content in barley (*Hordeum vulgare* L.) (Rustgi et al., 2019). However, the prolamins confer the viscoelastic properties of wheat dough that are required for the manufacture of many products, including bread, so this must also be considered when trying to breed for higher lysine content.

In maize, grain lysine content is similarly affected by the abundance of lysine‐poor proteins in the prolamin family called zeins. The expression of particular zein genes is determined by a bZIP transcription factor called *Opaque2* (Gavazzi et al., 2007), and the mutant line lacking a functional *Opaque2* gene is characterized by higher kernel lysine content (Mertz et al., 1964). Interestingly, the lysine QTL identified in this study is situated upstream of an *Opaque2* orthologue on chromosome 1A: TraesCS1A02G329900, otherwise known as storage protein activator (SPA), which is known to activate storage protein synthesis in wheat (Albani et al., 1997). The A genome homologue of SPA does not differ in sequence between Claire and Robigus, but differential expression of SPA (through differences in *TaHDT‐A1* regulation) is a possible mechanism by which this QTL could affect lysine content.

Future work investigating *TaHDT‐A1*, SPA, and other regulatory genes of storage proteins in wheat would help to elucidate their effects on grain lysine content and would be useful for expanding the germplasm available to increase lysine content, given the limited QTL and small effect of GS we found. Chromosome‐level assemblies of Claire and Robigus would also enable further analysis of this mapping population in the future. Combining both increased diversity and pangenomes, sequencing of the Watkins collection, and construction of genome assemblies will enable novel diversity to be identified that can be introgressed into elite soft wheat germplasm as well (Shewry et al., 2022).

## 5 CONCLUSIONS

The nutritional quality of the UK soft wheat can be improved incrementally using diversity from Claire and Robigus, but greater diversity is required to make larger gains. The genetic architecture of different amino acids differs considerably, and they are often controlled by QTL that impact other traits as well. Future soft wheat breeding in the UK should therefore consider use of more genetic diversity and using pleiotropic QTL to the benefit of farmers and consumers.

## AUTHOR CONTRIBUTIONS

**Joseph Oddy**: Conceptualization; Investigation; Writing‐original draft. **Monika Chhetry**: Investigation; Resources; Writing‐review & editing. **Rajani Awal**: Investigation; Resources; Writing‐review & editing. **John Addy**: Formal analysis; Investigation. **Mark Wilkinson**: Investigation; Supervision; Writing‐review & editing. **Dan Smith**: Formal analysis; Investigation; Writing‐review & editing. **Robert King**: Formal analysis; Investigation. **Chris Hall**: Investigation; Supervision. **Rebecca Testa**: Investigation. **Eve Murray**: Investigation. **Sarah Raffan**: Investigation; Supervision; Writing‐review & editing. **Tanya Y. Curtis**: Formal analysis; Investigation; Writing‐review & editing. **Luzie Wingen**: Formal analysis; Investigation; Supervision; Writing‐review & editing. **Simon Griffiths**: Formal analysis; Investigation; Writing‐review & editing. **Simon Berry**: Formal analysis; Investigation; Writing‐review & editing. **J. Stephen Elmore**: Supervision; Writing‐review & editing. **Nicholas Cryer**: Conceptualization; Project administration; Resources; Supervision; Writing‐review & editing. **Isabel Moreira de Almeida**: Conceptualization; Project administration; Resources; Supervision; Writing‐review & editing. **Nigel G. Halford**: Conceptualization; Funding acquisition; Project administration; Resources; Supervision; Writing‐review & editing.

## CONFLICT OF INTEREST STATEMENT

The authors declare no conflicts of interest.

## Supporting information

Supp Information
Supplemental Material
Supplementary figures – Supplementary figures 1 to 6.
Supplementary tables – Supplementary tables 1 to 6.
Supplementary data file 1 – R shiny QTL analysis files and data.
Supplementary data file 2 – HPC rrBLUP R markdown files.

## Data Availability

Data angenerated in this study are available from Dryad (https://doi.org/10.5061/dryad.b8gtht7hj).
