# Supplementary material for: Genetic control of grain amino acid composition in a UK soft wheat mapping population
Source: Plant Genome. 2023 May 3;16(4):e20335. doi: 10.1002/tpg2.20335 (PMC12806903; doi:10.1002/tpg2.20335)
Supplement: Supplementary file 1 [file TPG2-16-e20335-s001.pdf]

The Plant Genome OPEN ACCESS

Crop Science Society of America

Genetic control of grain amino acid composition in a UK soft wheat mapping population

Journal:	*The Plant Genome*
Manuscript ID	TPG-2022-07-0102.R1
Wiley - Manuscript type:	Original Article
Date Submitted by the Author:	23-Sep-2022

Complete List of Authors:	Oddy, Joseph; Rothamsted Research, Sustainable Soils and Crops<br>Chhetry, Monika; John Innes Centre<br>Awal, Rajani; John Innes Centre<br>Addy, John; Rothamsted Research<br>Wilkinson, Mark; Rothamsted Research<br>Smith, Dan; Rothamsted Research<br>King, Rob; Rothamsted Research<br>Hall, Chris; Rothamsted Research<br>Testa, Rebecca; Rothamsted Research<br>Murray, Eve; Rothamsted Research<br>Raffan, Sarah; Rothamsted Research<br>Curtis, Tanya; Curtis Analytics Ltd<br>Wingen, Luzie; John Innes Centre, Crop Genetics<br>Griffiths, Simon; John Innes Centre<br>Berry, Simon; Limagrain UK<br>Elmore, J. Stephen; University of Reading<br>Cryer, Nicholas; Mondelez UK Holdings and Services Ltd<br>Moreira de Almeida, Isabel; Mondelez European Biscuit Research and Development Saclay<br>Halford, Nigel G.; Rothamsted Research

Keywords:	Wheat, Seed physiology, Crop genetics

SCHOLARONE™
Manuscripts

Core Ideas

As part of the submission process, we ask authors to prepare highlights of their article. The highlights will consist of 3 to 5 bullet points that convey the core findings of the article and emphasize the novel aspects and impacts of the research on scientific progress and environmental problem solving.

The purpose of these highlights is to give a concise summary that will be helpful in assessing the suitability of the manuscript for publication in the journal and for selecting appropriate reviewers. If the article is accepted the highlights may also be used for promoting and publicizing the research.

Core Idea 1: â€¢ High free asparagine and low lysine concentrations limit the nutritional value of wheat grain.

Core Idea 2: â€¢ Investigation of a biparental mapping population formed from the UK soft wheats Claire and Robigus.

Core Idea 3: â€¢ Breeding for lower free asparagine and higher lysine using Claire and Robigus diversity is possible but limited.

Core Idea 4: CUST_CORE_IDEA_4 :No data available.

Core Idea 5: CUST_CORE_IDEA_5 :No data available.

For Review Only

Genetic control of grain amino acid composition in a UK soft wheat mapping population

Joseph Oddy[1], Monika Chhetry[2], Rajani Awal[2], John Addy[1], Mark Wilkinson[1], Dan Smith[1], Robert King[1], Chris Hall[1], Rebecca Testa[1], Eve Murray[1], Sarah Raffan[1], Tanya Y. Curtis[3], Luzie Wingen[2], Simon Griffiths[2], Simon Berry[4], J. Stephen Elmore[5], Nicholas Cryer[6], Isabel Moreira de Almeida[7], and Nigel G. Halford[1*]

[1]*Rothamsted Research, Harpenden, Hertfordshire AL5 2JQ, UK*

[2]*John Innes Centre, Norwich Research Park, Colney Lane, Norwich, UK*

[3]*Curtis Analytics Limited, Discovery Park, Sandwich, UK*

[4]*Limagrain UK Ltd, Market Rasen LN7 6DT, UK*

[5]*Department of Food & Nutritional Sciences, University of Reading, RG6 6DZ, UK*

[6]*Mondelēz UK R&D Ltd, Bournville Lane, Bournville, Birmingham, B30 2LU, UK*

[7]*Mondelēz R&D International, 6 Rue René Razel, 91400 Saclay, France*

For Review Only

Received:

Corresponding author (e-mail): nigel.halford@rothamsted.ac.uk

Abbreviations: QTL (quantitative trait locus/loci); GS (genomic selection); HFN (Hagberg falling number); KHI (kernel hardness index)

Running title: Genetic control of grain amino acid composition in a UK soft wheat mapping population

Core Ideas:

- High free asparagine and low lysine concentrations limit the nutritional value of wheat grain.
- Investigation of a biparental mapping population formed from the UK soft wheats Claire and Robigus.
- Breeding for lower free asparagine and higher lysine using Claire and Robigus diversity is possible but limited.

For Review Only

Abstract

Wheat (*Triticum aestivum* L.) is a major source of nutrients for populations across the globe, but the amino acid composition of wheat grain does not provide optimal nutrition. The nutritional value of wheat grain is limited by low concentrations of lysine (the most limiting essential amino acid) and high concentrations of free asparagine (precursor to the processing contaminant acrylamide). There are currently few available solutions for asparagine reduction and lysine biofortification through breeding. In this study, we investigated the genetic architecture controlling grain free amino acid composition and its relationship to other traits in a Robigus × Claire doubled haploid population. Multivariate analysis of amino acids and other traits showed that the two groups are largely independent of one another, with the largest effect on amino acids being from the environment. Linkage analysis of the population allowed identification of QTL controlling free amino acids and other traits, and this was compared against genomic prediction methods. Following identification of a QTL controlling free lysine content, wheat pangenome resources facilitated analysis of candidate genes in this region of the genome. These findings can be used to select appropriate strategies for lysine biofortification and free asparagine reduction in wheat breeding programmes.

For Review Only

1. Introduction

The nutritional quality of wheat (*Triticum aestivum* L.) has profound impacts on human health. As one of the largest sources of average daily calorie intake in the world (18.2% in 2019) (FAOSTAT, 2021), wheat is an essential source of macro and micronutrients. In 2019, 19.5% of average daily global protein intake was estimated to be provided by wheat-based foods (FAOSTAT, 2021). Similarly, between 2008 and 2017 in the UK, over 25% of average daily fibre intake was provided by wheat-based foods (Gressier & Frost, 2021). Wheat flour is often fortified to increase its nutrient content: in the UK, for example, wheat is fortified with calcium, iron, thiamine, niacin and, most recently, folate (DEFRA, 1998; DHSC, 2021). The quantities of different macro and micronutrients in wheat can have large impacts on population health because of the scale at which wheat products are consumed. For example, it is estimated that the addition of folate to UK flour will lead to a 20% decrease in neural tube defects in babies (DHSC, 2021). Consequently, it is essential to ensure that the nutritional profile of wheat is as beneficial as it can be for human health.

One way in which the nutritional profile of wheat can be improved is *via* optimisation of its amino acid composition, with the concentrations of lysine and asparagine most important. Free (soluble, non-protein) asparagine can be converted to the processing contaminant, acrylamide, during high-temperature cooking and processing, and this has led to ongoing efforts to reduce free asparagine concentration (Oddy *et al.*, 2022). Lysine, on the other hand, is not produced endogenously by humans or other monogastric animals, making it an essential amino acid in the diet, but it is present in only small quantities in wheat and other cereal grain and populations reliant on cereals for their nutrition may suffer from lysine deficiency (Galili & Amir, 2013). Indeed, fortifying wheat flour by adding lysine has been shown to improve indices of nutritional status in clinical trials in Pakistan, northern China, and Syria (Hussein *et al.*, 2004; Zhao *et al.*, 2004; Ghosh *et al.*, 2008). Flour fortification is unlikely to be a

For Review Only

sustainable solution in developing countries and it would be much cheaper and more efficient to increase the intrinsic lysine content of wheat grain. Therefore, the amino acid composition of wheat grain could be optimised both by decreasing grain free asparagine content and increasing lysine content.

In recent years, studies have investigated genetic strategies for the reduction of free asparagine content in wheat grain. Induced and natural variation in the asparagine synthetase 2 genes, for example, has been found to impact significantly on free asparagine content (Raffan *et al*., 2021; Oddy *et al*., 2021; Alarcon-Reverte *et al*., 2022) and quantitative trait loci (QTL) for grain asparagine content have been identified from previous GWAS studies (Emebiri, 2014; Peng et al., 2018; Rapp *et al*., 2018). However, the small number of stable QTL available to breeders limits the progress that can be made to reduce grain asparagine content in breeding programmes and no genetic strategies for soft (biscuit) wheat specifically have been investigated. Similarly, there are limited strategies currently available for increasing lysine content in wheat grain. Lysine biofortification *via* QTL identification and marker-assisted breeding has been studied extensively in both rice (*Oryza sativa* L.) (Wang *et al*., 2008; Zhong *et al*., 2011; Yoo, 2017; Jang *et al*., 2020) and maize (*Zea mays* L.) (Prasanna *et al*., 2020), but only two studies have previously investigated lysine biofortification in wheat through association studies. Peng et al. (2018) successfully identified QTL controlling free lysine and Jiang et al. (2013) identified QTL for total lysine.

Consequently, the aim of this study was to investigate QTL, genomic prediction accuracy, and candidate genes controlling the free amino acid composition of wheat grain in a soft wheat mapping population developed from the varieties Claire and Robigus. Like many UK varieties, these parents both lack the B genome homeologue of the asparagine synthetase-2 gene, *TaASN-B2* (TraesLDM3B03G01566640 in variety Landmark), the presence/absence of which is a known source of grain asparagine content variation (Oddy *et al*., 2021). This

For Review Only

mapping population, therefore, represents a useful resource for identifying additional variation. Claire and Robigus are also represented by scaffold-level genome assemblies in the wheat pangenome, facilitating candidate gene analysis. Furthermore, we investigated other traits, such as grain size, hardness, and Hagberg falling number (HFN), to determine whether QTL controlling nutritional traits overlapped with those controlling other traits of interest.

For Review Only

2. Materials and Methods

2.1. Production of Doubled Haploid lines

171 Doubled Haploid lines of Robigus x Claire were produced using a modified Knox *et al.* (2000) method. Wheat spikes were emasculated between growth stages GS55 and GS59. Once the stigma was receptive it was fertilised with freshly shed donor maize pollen. After one day, wheat florets were treated with Dicamba (20mgL^{-1}) (Sigma-Aldrich, D5417) and injected into the plant stem (100mgL^{-1}). Developing embryos were excised between 14 and 21 days. Under aseptic conditions, seeds were removed from the spikelets, surface sterilised with 70% (v/v) ethanol (EtOH) for 1 min, rinsed with sterile distilled water, and immersed in 20% (v/v) commercial bleach solution with a few drops of Tween® 20 for 20 mins. They were then rinsed with sterile distilled water three times.

For Review Only

Haploid embryos were excised and grown on 90mm Petri dishes in the dark on Gamborg's B5 media with minimal organics (Gamborg *et al.*, 1968), 2% (w/v) sucrose, pH 5.8, 9gL^{-1} Difco bactoagar at 20°C. When showing signs of germination, embryos were transferred to a light incubator at 20°C. Any non-germinated 1 month old embryos were given cold shock treatment at 4°C for 7 days to promote germination. Germinated plantlets were vernalised for 4 weeks and were grown in the glasshouse until the 4-tiller stage. Plants were then given colchicine (Sigma-Aldrich, C9754) treatment for 5 to 6 hours in the light at room temperature, washed and transplanted to soil, acclimatised and grown in a glasshouse. The mapping population was genotyped by Limagrain using a proprietary SNP array. The genetic map comprising 872 loci was constructed using MSTMap Online (http://mstmap.org/).

The mapping population was grown in field trials at the John Innes Centre Morley Mill Hill field site (52°33′15.1″N 1°01′59.2″E) in 2017 to 2018 (abbreviated as H18) and at the Church Farm field site (52°38′N 1°10′E) in 2018 to 2019 (abbreviated as H19). All 171 Doubled Haploid lines of the mapping population were grown in each trial. Within each trial,

one replicate of each line was drilled in 6 m^2 plots in a completely randomised design. The H18 field trial was drilled on the 21st September 2017 and harvested on the 1st August 2018. The H19 field trial was drilled on the 14th September 2018 and harvested on the 12th August 2019. Growth habit, heading date, plant height, and yield traits were scored in the field.

DoubleTop fertiliser (27N 30SO_3) was applied at a rate of 150 kg/ha on the 20th March 2018 for H18 and the 23rd February 2019 for H19. In both trials, slug control pellets were applied at a rate of 7 kg/ha after drilling to control slug pests (Gusto (metaldehyde) pellets on 27th September 2017 for H18 and Sluxx (Ferric phosphate) pellets on 24th September 2018 for H19). Herbicide mixtures were applied in autumn (21st November 2017 for H18 and 24th September 2018 for H19) and spring (23rd May 2018 for H18 and 19th March 2019 for H19) for both trials to control weeds.

2.2. Phenotyping

Grain diameter, kernel hardness index (KHI), and grain weight measurements were recorded for 300 kernels from each line in the population using a Perten Single Kernel Classification System (SKCS) 4100 (Calibre Control International Ltd., Warrington, UK). Grain length (mm), width (mm), and area (mm^2) measurements were recorded in triplicate for each sample using a MARVIN Seed Analyser and software Marvin 4.0 (MARViTECH GmbH, Wittenburg, Germany). Grain samples were milled to wholemeal flour in a coffee grinder and flour moisture content was recorded using a Minispec nuclear magnetic resonance (NMR) analyser (Minispec Mq10, Bruker Inc., Germany). Hagberg falling number measurements were recorded using an FN 1000 as the average of two technical replicates (Perten, Sweden), adjusting for flour moisture content as required according to manufacturer's instructions. Amino acid analysis was performed on wholemeal flour samples by HPLC as described previously (Raffan *et al.*,

For Review Only

2021) by Curtis Analytics (Sandwich, UK). Briefly, free amino acids were extracted from 0.5g of wholemeal flour and underwent precolumn derivatisation (Curtis et al., 2018). Samples were then run on an HPLC system identically to previously described (Raffan et al., 2021). Three technical replicates were taken for each sample for amino acid measurement.

2.3. Phenotypic data analysis

Skewness and kurtosis were measured for all variables in each environment and normal plots visually inspected in Genstat (VSN International, 2021) to determine if variables required transformation. The data were appropriately transformed according to their distribution if necessary (see Tables S1 and S2 for details of transformations). Subsequent analyses were performed on transformed variables unless otherwise stated. Plotting was performed in R (R Core Team, 2021) with the packages ggplot2 (Wickham, 2016), tidyverse (Wickham et al., 2019), and cowplot (Wilke, 2020).

Broad-sense heritability for each trait was estimated as described in Covarrubias-Pazaran (2019) using the packages dplyr (Wickham et al., 2022) and lme4 (Bates et al., 2015). Kendall rank correlation coefficients were performed on non-transformed data and adjusted p values (Bonferroni correction) were calculated for plotting using R (R Core Team, 2021) and the package corrplot (Wei & Simko, 2021). Principal component analysis was performed on untransformed, scaled variables using the package factoextra (Kassambara and Mundt, 2020). Correlation network analysis was performed and plotted by filtering for significant correlations where $p < 0.001$ using Kendall correlation with Bonferroni correction using the packages corrr (Kuhn, Jackson and Cimentada, 2020), igraph (Csardi and Nepusz, 2006), and ggraph (Pedersen, 2021).

Bayesian modelling was performed on untransformed variables in R using the package rstanarm (Goodrich *et al*., 2020). Variables were scaled before modelling and individual linear

For Review Only

models for each predictor variable were created to guide the selection of informative priors. Simulations of the posterior distribution were subsequently performed to check model fit and intervals were plotted using the package bayesplot (Gabry and Mahr, 2022). R^2 estimates were obtained by taking the median of leave-one-out cross validation adjusted estimates.

2.4. Linkage analysis

Single-environment linkage analysis was performed in R using packages qtl (Broman *et al*., 2003) and qtl2 (Broman *et al*., 2018). Single-environment linkage analysis was made into an interactive app using the packages shiny (Chang *et al*., 2021), plyr (Wickham, 2011), and rsconnect (Atkins, McPherson & Allaire, 2021), accessible at https://t9onwp-wheatworker.shinyapps.io/QTL_Browser/ and in supplementary data file 1. As before, SIM was performed first to identify covariates for use in CIM. Identified QTL from CIM were then used to create single QTL models as well as additive QTL models. Upper and lower 95% confidence intervals for QTL location were calculated using the Bayesian credible interval method in R/qtl and expanded to the closest markers. Pseudomarkers were generated every 2 cM in the map and the minimum marker covariate proximity was set at 20 cM. A logarithm of the odds (LOD) score of 3 was used as the significance threshold.

Multi-environment single trait linkage analysis was performed in Genstat for each trait to detect QTL present in both environments, following selection of the most appropriate variance-covariance model according to the Bayesian information criterion. Simple interval mapping (SIM) was initially performed to identify putative QTL. These QTL were then used as covariates in composite interval mapping (CIM). QTL identified from CIM were then used to construct the final QTL models. Pseudo-markers were generated every 2 cM in the map. The minimum cofactor proximity was set at 30 cM and the minimum separation for selected QTL

For Review Only

at 20 cM. Significance thresholds were determined by the Li and Ji method (Li & Ji, 2005) with a genome-wide significance level of 0.05.

2.5. Genomic prediction

Genomic prediction was performed for each trait *via* five-fold cross validation with 10,000 permutations using the R package rrBLUP (Endelman, 2011). The "mixed.solve" function within this package was used to estimate marker effects for each trait, with the identity matrix being left unspecified. Pearson correlation coefficients were calculated for the results from the training and testing datasets to estimate genomic prediction accuracy. For within year prediction estimates, training and testing datasets came from the same trial. For between year prediction estimates, training and testing datasets were from different trials. Further detail is available as R markdown in supplementary data file 2. Scripts were submitted to the high-performance computing cluster at Rothamsted Research via SLURM for execution.

2.6. Candidate gene analysis

The gene content of the lysine QTL was determined for all wheat pangenome varieties at chromosome scale assembly by identifying the location of the markers in these varieties and extracting genes from Ensembl Biomart (Howe et al., 2021). Genes residing within the region in variety Chinese Spring v1.0 were submitted to KnetMiner (https://knetminer.com/Triticum_aestivum/) (Hassani-Pak *et al.*, 2021) for ranking on relevant keywords ("Lysine", "Storage proteins"). Expression of the top hits was then investigated in expVIP (Borrill, Ramirez-Gonzalez, & Uauy, 2016) to further narrow down plausible candidate genes. Transcript per million (TPM) data for the Azhurnaya developmental time-course experiment were extracted from expVIP for plotting in R using the package pheatmap (Kolde, 2019). Corresponding Claire and Robigus genes were then identified from these

For Review Only

Chinese Spring candidate genes in Ensembl and pairwise aligned via BLAST using Geneious Prime 2020.1.2 to identify variation.

For Review Only

3. Results

3.1. Phenotypic analysis

We measured free amino acid concentrations and other traits of interest in the Robigus × Claire mapping population from field trials grown in 2017–2018 (H18) and 2018–2019 (H19) (Figure 1; Figure S1). Aspartic acid, asparagine, and glutamic acid were the most abundant of the free amino acids measured, with concentrations of free amino acids consistently higher in H19 than in H18 (Figure 1a). Principal component analysis revealed harvest year to be a key driver of variation in this dataset (Figure 1b) and, notably, the second harvest year (H19) also showed lower yield alongside the increased free amino acid content of the grain (Figure 1b). PCA and correlation network analysis revealed that most of the other traits measured here were uncorrelated with the amino acids (Figure 1b; Figure 1c; Figure S2; Figure S3), except for grain yield which showed negative correlations with a subset of amino acids (Figure 1b; Figure 1c; Figure 2a).

To understand whether any of the traits we measured could predict free asparagine or lysine content in the grain, we constructed Bayesian linear models with the traits and harvest year as explanatory variables (Figure 2b; Figure 2c). In both the free asparagine (Figure 2b) and lysine (Figure 2c) models, environment had the greatest effect whereas other variables had little explanatory power. Nevertheless, the variance explained in the models was still reasonable for asparagine at 56.5%, but only 22.2% for lysine.

3.2. QTL analysis

Broad-sense heritability estimates varied substantially between the different amino acids, with free asparagine and lysine showing heritability estimates of 0.60 and 0.45, respectively (Table S1). Aspartic acid showed the highest heritability of the amino acids measured here, with an estimate of 0.82. Heritability estimates for the size traits were generally very high, as expected,

For Review Only

and correlation of these values between years was also stronger than the correlation of amino acids between years (Table S1).

We identified QTL for grain free asparagine content and lysine content on chromosomes 4B and 1A, respectively (Figure 3a; Figure 3b; Table 1), which had significant effects across both environments but were also affected by QTL by environment effects (Figure 3c; Figure 3d; Table 1; Table S2). The asparagine QTL on 4B explained 2.6% of the variance in H18, when free asparagine concentrations were lower overall, whereas it explained 14.8% of the variance in H19, when free asparagine concentrations were elevated (Table 1). In both years, the Robigus allele was associated with the higher free asparagine concentrations. In contrast, the lysine QTL on 1A explained 12.1% of the variance in H18, when free lysine was lower overall, and only 2.6% of the variance in H19, when free lysine concentrations were elevated. The Claire allele was associated with higher free lysine concentrations in both years in this case. Multi-environment linkage analysis of amino acid and grain measurements revealed many QTL controlling the other amino acids and traits as well (Table 1; Table S2; Table S3).

For Review Only

The QTL controlling asparagine on chromosome 4B appeared to overlap with QTL for glutamine, glutamic acid, and glycine (Table 1). Each of these QTL had a greater effect in H18 than in H19 and the Robigus allele was associated with the higher value in each case as well, suggesting that these QTL are caused by the same variant. These QTL were also located near to a QTL for KHI and more distantly to QTL for grain diameter, plant height, and grain weight (Table 2, Figure S4), which are likely caused by the *Rht-B1* polymorphism. QTL for aspartic acid also appeared to overlap with QTL for other traits (Table 2). For aspartic acid on 4A and 4D, there are co-locating HFN QTL, suggesting that these two traits are under the control of

the same locus (Figure S5). The location of the QTL on 4D matches the *Rht-D1* polymorphism between Claire and Robigus found at 18.78 Mbp in Chinese Spring. Of all the amino acids measured in this study, we identified the most QTL controlling aspartic acid (Table 1). Other potential sources of variation underpinning the QTL in this study are presented in Table S4.

3.3. Genomic prediction

Following our modelling of asparagine and lysine using agronomic measurements and QTL models, we calculated the accuracy of genomic prediction (GP) for within and between year prediction of traits (Figure 4a; Figure S6; Table S1). Prediction accuracy was more consistent when performed across years rather than within years (Figure S6), so these were used for further interpretation. Prediction accuracy for lysine was the lowest of all traits at a mean accuracy of 0.10, whereas accuracy for asparagine was around 0.34. Of all amino acids, aspartic acid had the greatest prediction accuracy results. Prediction accuracies for the other functional traits were generally higher than the accuracies for amino acids, as expected from the higher heritability of these traits. Comparing the amount of variation explained by genomic prediction methods and additive QTL models, we can see that the GP models explain more variance than the additive QTL models for all traits (Figure 4b).

For Review Only

3.4. Lysine QTL candidate gene analysis

The gene content and QTL size of the lysine QTL on 1A, the HFN/aspartic acid/KHI QTL on 4A, and the asparagine QTL on 4B differed substantially (Table S5). Due to the size of the 4A and 4B QTL, we were unable to plausibly narrow down candidate genes, whereas the lysine QTL on 1A was much smaller so amenable to further analysis. We investigated the gene content of the lysine QTL for all genomes assembled to chromosome scale in the wheat pangenome and gene content varied to a small extent between the different varieties (Table

S6). Most notably, the QTL did not match any locations in variety Julius and matched to an unanchored scaffold in Stanley.

KnetMiner analysis of the genes residing in Chinese Spring in the lysine QTL was undertaken with relevant keywords to highlight possible candidate genes, and these genes were subsequently investigated for their expression patterns from expVIP. Pairwise analysis of the top KnetMiner hits in the lysine QTL showed that the top hit (TRAESCS1A02G445700) differed between Claire and Robigus. TRAESCS1A02G445700, or *TaHDT-A1*, has been identified as a member of the histone deacetylase family in wheat. A deletion within the CDS of the gene in Robigus means that the most highly expressed transcript cannot be expressed (Figure 5) and the two missing exons from this most highly expressed transcript form a zinc finger/C2H2 DNA binding domain, which is important for transcriptional regulation.

For Review Only

4. Discussion

4.1. Limited variation in Claire and Robigus for asparagine and lysine improvement

Soft wheat breeding in the UK has relied heavily upon Claire and Robigus as parents since their development in 1999 and 2005, respectively. A recent study found that UK winter wheats developed between 2002 and 2017 could be clustered into four distinct populations, and two of these populations were characterised by their Claire or Robigus heritage (Shorinola et al, 2022). The varieties within these population groups characterised by Claire and Robigus heritage are also almost entirely soft wheat varieties, further emphasising the importance of these two varieties in UK soft wheat breeding. This large contribution of Claire and Robigus as parents to soft wheat breeding means that opportunities for nutritional improvement have often been limited to variation between these two parents.

Our analysis found that there is variation between Claire and Robigus and that this does impact asparagine and lysine content to a small extent. Asparagine had a moderate heritability (0.60) across both field trials in the study, whereas the heritability for lysine was lower (0.45). One QTL was found for asparagine and lysine each, both explaining less than 10% of the variance on average. The asparagine QTL identified here (peak at 601.4 Mbp in Chinese Spring) lies around 60 Mbp from another QTL (peak at 660.7 Mbp in Chinese Spring) identified by Peng et al. (2018), suggesting that these may coincide, whereas the lysine QTL does not overlap with previously identified QTL. Genomic selection had a predictive ability of 0.34 on average for asparagine, indicating that this method may be better suited for breeding because of the genetic architecture of this trait (many small-effect QTL). Rapp et al. (2018) also found that GS had a predictive ability of around 0.5 on average for asparagine, the higher estimate in this study likely due to within environment prediction and analysis of a more diverse mapping population. GS only achieved a predictive ability of 0.10 for lysine, indicating that

For Review Only

only incremental advances in lysine content are possible using Claire and Robigus. Previous GWAS studies using more diverse panels have found more, larger-effect QTL controlling asparagine and lysine content (Peng et al., 2018; Rapp et al., 2018), indicating that there may be beneficial alleles in more diverse germplasm. Consequently, UK soft wheat germplasm will require diversity beyond Claire and Robigus to make changes to asparagine and lysine content beyond the incremental improvements found here.

4.2. Trade-offs between amino acid content and other traits

Another aspect we wanted to investigate in this population was whether there were any relationships between amino acids and other traits. Amino acids tended to correlate positively with one another and were mostly unrelated to the other measured traits, with the exception of grain yield and kernel hardness index. A negative correlation between grain yield and free asparagine has previously been documented (Xie et al., 2021), but in other experiments the association has been positive (Malunga et al., 2021; Xie et al., 2021). In our analysis, this association mostly arose because of the effect of environment on both yield and asparagine. Environmental stress can lead to decreases in yield whilst increasing free asparagine, whilst other variables (e.g., nitrogen fertiliser) can lead to increases in both yield and free asparagine (see Oddy et al. (2022) for review). Our modelling of asparagine through these variables mostly indicated environment as the driving force in our study, but there was still a slight negative association with yield and plant height as well as a slight positive association with kernel hardness. Kernel hardness, like grain free asparagine content, is known to increase with nitrogen application, which may underly this small association with asparagine.

A strong environmental effect on free asparagine concentration has been observed in response to many different stressors (see Oddy et al., 2020 for review) and it is under stressful conditions that the highest asparagine levels are often observed. These increases in grain

For Review Only

asparagine concentration vary massively, causing unexpected blips in acrylamide content in food products. These environmentally induced increases pose the greatest threat to food safety and regulatory compliance, so elimination of this environmental response would be of great interest. A weak environmental effect was seen in this study: during the 2018–2019 season the average amino acid concentrations rose whilst the yields dropped. Interestingly, the asparagine QTL we identified here had greater effect in this season, enabling reductions of 15.68% in free asparagine concentrations in those lines possessing the Claire allele over those possessing the Robigus allele. This suggests that this QTL may be more effective under more stressful conditions, so selection of the Claire allele at this locus may prove beneficial for reducing the large free asparagine increases observed following environmental stress. This is in contrast to the effect of the *TaASN-B2* deletion, which has a greater effect when grain asparagine concentrations are lower (Oddy et al., 2021), when plants are not suffering from sulphur deficiency. Future work would therefore benefit from identification of similar QTL that are associated with lowering asparagine content from the high levels seen during stress. This would enable the stacking of alleles that are beneficial under both stress and non-stress conditions, to ensure that free asparagine concentrations are minimised in all environments.

We also wanted to understand whether any QTL controlling amino acid content had pleiotropic effects on other traits. The asparagine QTL we identified on chromosome 4B appeared to overlap with QTL for plant height in the first year, suggesting that there might be an impact of the *Rht-B1b* allele on asparagine. The *Rht* genes are dwarfing genes used during the green revolution that have many impacts on crop traits beyond height (Casebow et al., 2016) and Claire and Robigus both possess different *Rht* genes on 4B and 4D (Wilkinson et al., 2020). However, this QTL overlap was not present in the second year of analysis when the asparagine QTL had a greater effect, suggesting that the QTL controlling height and asparagine may be distinct. However, a more detailed analysis is required to comprehensively assess the

For Review Only

impact of *Rht-B1* alleles (and dwarfing genes in general) on grain asparagine content. The QTL controlling asparagine did overlap consistently with a QTL for KHI though, with the 'increasing allele' belonging to Robigus for both traits. Kernel hardness and free asparagine content are both known to correlate under certain conditions with nitrogen content (Oddy et al., 2022), so this QTL may be linked to nitrogen use efficiency/uptake. The KHI QTL on 4B also exhibited a similar genotype by environment effect pattern to the asparagine QTL, with a greater effect of the QTL observed in the second trial year. Selection for the Claire allele at this QTL would therefore be suitable in the context of soft wheat breeding, where both softer textures and lower asparagine content are desirable.

Interestingly, we found much more genetic control of free aspartic acid concentration in this population compared to the other amino acids. Heritability was high (> 0.8), genomic prediction accuracy was moderate (> 0.5, same as grain weight), and there were four multi-environment QTL controlling the trait. Two of the QTL controlling aspartic acid also overlapped with QTL controlling HFN. One of these QTL was situated on 4D and overlapped with traits for plant height and grain size as well, indicating that this may be due to *Rht-D1* allele status, which is known to impact HFN as well as plant height (Fradgley et al., 2022). The second QTL controlling both aspartic acid and HFN was situated on 4A and also overlapped with traits for grain size and KHI. Previous work has identified a major QTL underlying pre-harvesting sprout (PHS) variation on 4A, but both Claire and Robigus share the same *MKK3-A* allele which underlies this QTL (Shorinola et al., 2017). Li *et al*. (2021) also identified a PHS QTL in a similar region on 4A but this does not overlap with the region identified here. One possible source of variation underlying the QTL controlling aspartic acid and HFN on 4A is the *Triticum dicoccoides* introgression in Robigus, which matches the region this QTL is found in (Przewieslik-Allen et al., 2021). The antagonistic relationship between HFN and asparagine

For Review Only

at this QTL could be a result of increased HFN reducing proteolysis, and thereby preventing accumulation of free amino acids.

4.3. Lysine candidate genes

Scaffold-level genome assemblies of Claire and Robigus (Walkowiak et al., 2020) enabled us to investigate the lysine QTL in greater depth, identifying the candidate gene *TaHDT-A1*, encoding a histone deacetylase. The wheat histone deacetylase family is very large, encompassing approximately 50 genes (Jin et al., 2020, Li et al., 2022). Histone deacetylases function mainly to inhibit gene expression because histone deacetylation causes chromatin condensation, with roles in many different developmental processes and environmental responses. In wheat, it is known that differences in grain lysine content can be caused by differential expression of lysine-poor storage proteins (prolamins). Gill-Humanes *et al*. (2014), for example, identified downregulation of gliadins (a class of prolamins) as a method of increasing lysine content in wheat, and Moehs *et al*. (2019) showed that mutation of wheat prolamin binding factor (*WPBF*), a DOF-class transcription factor, increased lysine concentration. Lower prolamin protein content is also associated with increased lysine content in barley (*Hordeum vulgare* L.) (Rustgi *et al*., 2019). However, the prolamins confer the viscoelastic properties of wheat dough that are required for the manufacture of many products, including bread, so this must also be considered when trying to breed for higher lysine content.

In maize, grain lysine content is similarly affected by the abundance of lysine-poor proteins in the prolamin family called zeins. The expression of particular zein genes is determined by a bZIP transcription factor called *Opaque2* (Gavazzi et al., 2007), and the mutant line lacking a functional *Opaque2* gene is characterised by higher kernel lysine content (Mertz et al., 1964). Interestingly, the lysine QTL identified in this study is situated upstream of an *Opaque2* orthologue on chromosome 1A: TraesCS1A02G329900, otherwise known as *SPA*

For Review Only

(storage protein activator), which is known to activate storage protein synthesis in wheat (Albani et al., 1997). The A genome homeologue of *SPA* does not differ in sequence between Claire and Robigus, but differential expression of *SPA* (through differences in HDT1 regulation) is a possible mechanism by which this QTL could affect lysine content.

Future work investigating *HDT1*, *SPA*, and other regulatory genes of storage proteins in wheat would help to elucidate their effects on grain lysine content and would be useful for expanding the germplasm available to increase lysine content, given the limited QTL and small effect of GS we found. Chromosome-level assemblies of Claire and Robigus would also enable further analysis of this mapping population in the future. Combining both increased diversity and pangenomes, sequencing of the Watkins collection and construction of genome assemblies will enable novel diversity to be identified that can be introgressed into elite soft wheat germplasm as well (Shewry et al., 2022).

5. Conclusions

The nutritional quality of UK soft wheat can be improved incrementally using diversity from Claire and Robigus, but greater diversity is required to make larger gains. The genetic architecture of different amino acids differs considerably, and they are often controlled by QTL that impact other traits as well. Future soft wheat breeding in the UK should therefore consider use of more genetic diversity and using pleiotropic QTL to the benefit of farmers and consumers.

For Review Only

Author Contributions

Joseph Oddy: Data collection, computational analysis, writing. **Monika Chhetry:** Generation of mapping population. **Rajani Awal:** Generation of mapping population. **John Addy:** Statistical guidance. **Mark Wilkinson:** Project planning, supervision. **Dan Smith:** Bioinformatic guidance. **Robert King:** Bioinformatic guidance. **Chris Hall:** Data collection guidance. **Rebecca Testa:** Data collection. **Eve Murray:** Data collection. **Sarah Raffan:** Project planning. **Tanya Curtis:** Data collection. **Luzie Wingen:** Statistical guidance. **Simon Griffiths:** Project planning. **Simon Berry:** Generation of genotyping data. **J. Stephen Elmore:** Project planning, supervision. **Nicholas Cryer:** Project planning, supervision. **Isabel Moreira de Almeida:** Project planning, supervision. **Nigel G. Halford:** Project planning, supervision, writing.

Acknowledgements

JO is supported by a BBSRC Collaborative Training Partnership Studentship (BB/T50838X/1) with partners: University of Reading and Mondelez UK R&D Ltd. JO is also supported by a scholarship from the Society of Chemical Industry. NGH is supported at Rothamsted Research by the BBSRC *via* the Designing Future Wheat Programme (BB/P016855/1). SR is supported by a Biotechnology and Biological Sciences Research Council (BBSRC) Super Follow-on Fund grant (BB/T017007/1), with partners: University of Bristol, AHDB, KWS UK Ltd, Saaten Union UK Ltd, RAGT Seeds Ltd, Syngenta UK Ltd, and Limagrain UK Ltd. BBSRC is part of UK Research and Innovation. Many thanks to Steve Hanley, Steve Thomas, Andy Phillips, Friedrich Longin, Matthias Rapp, and Keywan Hassani-Pak for their discussions and help in this study.

Conflict of Interest

For Review Only

Joseph Oddy is supported by a BBSRC Collaborative Training Partnership Studentship with partners: University of Reading and Mondelez UK R&D Ltd. JO is also supported by a scholarship from the Society of Chemical Industry. Sarah Raffan is supported by a Biotechnology and Biological Sciences Research Council (BBSRC) Super Follow-on Fund grant, with partners: University of Bristol, AHDB, KWS UK Ltd, Saaten Union UK Ltd, RAGT Seeds Ltd, Syngenta UK Ltd, and Limagrain UK Ltd.

Supplemental Material

Supplementary figures – Supplementary figures 1 to 6.

Supplementary tables – Supplementary tables 1 to 6.

Supplementary data file 1 – R shiny QTL analysis files and data.

Supplementary data file 2 – HPC rrBLUP R markdown files.

Data availability

Data generated in this study are available from Dryad (doi:10.5061/dryad.b8gtht7hj).

For Review Only

References

Alarcón-Reverte, R., Xie, Y., Stromberger, J., Cotter, J.D., Mason, R.E., & Pearce, S. (2022). Induced mutations in ASPARAGINE SYNTHETASE-A2 reduce free asparagine concentration in the wheat grain. *Crop Science*, https://doi.org/10.1002/csc2.20760

Albani, D., Hammond-Kosack, M.C.U., Smith, C., Conlan, S., Colot, V., Holdsworth, M., & Bevan, M.W. (1997). The wheat transcriptional activator SPA: A seed-specific bZIP protein that recognizes the GCN4-like motif in the bifactorial endosperm box of prolamin genes. *Plant Cell*, https://doi.org/10.1105/tpc.9.2.171

Atkins, A., McPherson, J., & Allaire, J.J. (2021). rsconnect: Deployment Interface for R Markdown Documents and Shiny Applications. R package version 0.8.25. https://CRAN.R-project.org/package=rsconnect

Bates, D., Maechler, M., Bolker, B., and Walker, S. (2015). Fitting Linear Mixed-Effects Models Using lme4. *Journal of Statistical Software*, 67(1), 1-48. doi:10.18637/jss.v067.i01.

Borrill, P., Ramirez-Gonzalez, R., & Uauy, C. (2016). expVIP: a customizable RNA-seq data analysis and visualization platform. *Plant Physiology*, 170, 2172–2186.

Broman, K.W., Gatti, D.M., Simecek, P., Furlotte, N.A., Prins, P., Sen, Ś., Yandell, B.S., & Churchill, G.A. (2019). R/qtl2: software for mapping quantitative trait loci with high-dimensional data and multiparent populations. *Genetics*, *211*, 495–502.

Broman, K.W., Wu, H., Sen, Ś., & Churchill, G.A. (2003). R/qtl: QTL mapping in experimental crosses. *Bioinformatics*, *19*, 889–890.

Casebow, R., Hadley, C., Uppal, R., Addisu, M., Loddo, S., Kowalski, A., Griffiths, S., & Gooding, M. (2016). Reduced height (Rht) alleles affect wheat grain quality. *PloS one*, *11*, e0156056

Chang, W., Cheng, J., Allaire, JJ., Sievert, C., Schloerke, B., Xie, Y., Allen, J., McPherson, J., Dipert, A.,& Borges, B. (2021). shiny:Web Application Framework for R. R package version 1.7.1. https://CRAN.R-project.org/package=shiny

Csardi, G., Nepusz, T. (2006) The igraph software package for complex network research, InterJournal, Complex Systems 1695. https://igraph.org

Curtis, T.Y., Powers, S.J., Wang, R., & Halford, N.G. (2018). Effects of variety, year of cultivation and sulphur supply on the accumulation of free asparagine in the grain of commercial wheat varieties. *Food Chemistry*, https://doi.org/10.1016/j.foodchem.2017.06.113

DEFRA (1998). The Bread and Flour Regulations 1998. https://www.legislation.gov.uk/uksi/1998/141/contents/made (accessed September 16, 2022)

Department of Health and Social Care UK Government. (2021). *Folic Acid Added to Flour to Prevent Spinal Conditions in Babies*. https://www.gov.uk/government/news/folic-acid-added-to-flour-to-prevent-spinal-conditions-in-babies (accessed February 24, 2022).

Emebiri, L.C. (2014). Genetic variation and possible SNP markers for breeding wheat with low-grain asparagine, the major precursor for acrylamide formation in heat-processed products. *Journal of the Science of Food and Agriculture*. https://doi.org/10.1002/jsfa.6434

For Review Only

Endelman, J.B. (2011). Ridge regression and other kernels for genomic selection with R package rrBLUP. *The Plant Genome*, *4*, 250–255. https://doi.org/10.3835/plantgenome2011.08.0024

FAO. (2021). *FAOSTAT: Food Balances (2014-)*. http://www.fao.org/faostat/en/ (accessed February 1, 2022).

Fradgley, N.S., Gardner, K., Kerton, M., Swarbreck, S.M., & Bentley, A.R. (2022). Trade-offs in the genetic control of functional and nutritional quality traits in UK winter wheat. *Heredity*, *128*, 420–433. https://doi.org/10.1038/s41437-022-00503-7

Gabry J, Mahr T (2022). "bayesplot: Plotting for Bayesian Models." R package version 1.9.0, <URL: https://mc-stan.org/bayesplot/>.

Galili, G., & Amir, R. (2013). Fortifying plants with the essential amino acids lysine and methionine to improve nutritional quality. *Plant Biotechnology Journal*, *11*, 211–222

Gamborg, O.L., Miller, R., Ojima, K. (1968). Nutrient requirements of suspension cultures of soybean root cells. *Experimental Cell Research*, 50, 151–158.

Gavazzi, F., Lazzari, B., Ciceri, P., Gianazza, E., & Viotti, A. (2007). Wild-type opaque2 and defective opaque2 polypeptides form complexes in maize endosperm cells and bind the opaque2-zein target site. *Plant Physiology*, *145*, 933–945

Gil-Humanes, J., Pistón, F., Altamirano-Fortoul, R., Real, A., Comino, I., Sousa, C., Rosell, C.M., & Barro, F. (2014). Reduced-gliadin wheat bread: an alternative to the gluten-free diet for consumers suffering gluten-related pathologies. *PloS one*, *9*, e90898

For Review Only

Covarrubias-Pazaran, G. (2019). *Heritability: Meaning and Computation*. https://excellenceinbreeding.org/sites/default/files/manual/EiB-M2_Heritability_18-02-20.pdf (accessed March 1, 2022).

Goodrich, B., Gabry, J., Ali, I., & Brilleman, S. (2020). rstanarm: Bayesian applied regression modeling via Stan. R package version 2.21.1 https://mc-stan.org/rstanarm.

Gressier, M., & Frost, G. (2022). Minor changes in fibre intake in the UK population between 2008/2009 and 2016/2017. *European Journal of Clinical Nutrition*, *76*, 322–327

Hassani-Pak, K., Singh, A., Brandizi, M., Hearnshaw, J., Parsons, J.D., Amberkar, S., Phillips, A.L., Doonan, J.H., & Rawlings, C. (2021). KnetMiner: a comprehensive approach for supporting evidence-based gene discovery and complex trait analysis across species. *Plant Biotechnology Journal*, *19*, 1670–1678

Howe, K.L., Achuthan, P., Allen, J., Allen, J., Alvarez-Jarreta, J., Amode, M.R., Armean, I.M., Azov, A.G., Bennett, R., & Bhai, J. (2021). Ensembl 2021. *Nucleic Acids Research*, *49*, D884–D891

Jang, S., Han, J.-H., Lee, Y.K., Shin, N.-H., Kang, Y.J., Kim, C.-K., & Chin, J.H. (2020). Mapping and validation of QTLs for the amino acid and total protein content in brown rice. *Frontiers in Genetics*, *11*, 240

Jiang, X., Deng, Z., Ru, Z., Wu, P., & Tian, J. (2013). Quantitative trait loci controlling amino acid contents in wheat (*Triticum aestivum* L.). *Australian Journal of Crop Science*, *7*, 820–829

For Review Only

Jin, P., Gao, S., He, L., Xu, M., Zhang, T., Zhang, F., Jiang, Y., Liu, T., Yang, J., & Yang, J. (2020). Genome-wide identification and expression analysis of the histone deacetylase gene family in wheat (*Triticum aestivum* L.). *Plants*, *10*, 19

Kassambara, A., & Mundt, F. (2020). factoextra: Extract and Visualize the Results of Multivariate Data Analyses. R package version 1.0.7. https://CRAN.R-project.org/package=factoextra

Knox, R.E., Clarke, J.M., & DePauw, R.M. (2000). Dicamba and growth condition effects on doubled haploid production in durum wheat crossed with maize. *Plant Breeding*, 119, 289–298

Kolde, R. (2019). pheatmap: Pretty Heatmaps. R package version 1.0.12. https://CRAN.R-project.org/package=pheatmap

Kuhn, M., Jackson, S., and Cimentada, J. (2020). corrr: Correlations in R. R package version 0.4.3. https://CRAN.R-project.org/package=corrr

Li, H., Liu, H., Pei, X., Chen, H., Li, X., Wang, J., & Wang, C. (2021). Comparative Genome-Wide Analysis and Expression Profiling of Histone Acetyltransferases and Histone Deacetylases Involved in the Response to Drought in Wheat. *Journal of Plant Growth Regulation*, *41*, 1065-1078.

Li, L., Zhang, Y., Zhang, Y., Li, M., Tian, X., Song, J., Luo, X., Xie, L., Wang, D., & He, Z. (2021). Genome-Wide Linkage Mapping for Preharvest Sprouting Resistance in Wheat Using 15K Single-Nucleotide Polymorphism Arrays. *Frontiers in Plant Science*, *12*, 749206. https://doi.org/10.3389/fpls.2021.749206

For Review Only

Malunga, L.N., Ames, N., Khorshidi, A.S., Thandapilly, S.J., Yan, W., Dyck, A., Waterer, J., Malcolmson, L., Cuthbert, R., Sopiwnyk, E., & Scanlon, M.G. (2021). Association of asparagine concentration in wheat with cultivar, location, fertilizer, and their interaction. *Food Chemistry*, *344*. https://doi.org/10.1016/j.foodchem.2020.128630

Mertz, E.T., Bates, L.S., & Nelson, O.E. (1964). Mutant gene that changes protein composition and increases lysine content of maize endosperm. *Science*, *145*, 279–280

Moehs, C.P., Austill, W.J., Holm, A., Large, T.A.G., Loeffler, D., Mullenberg, J., Schnable, P.S., Skinner, W., van Boxtel, J., & Wu, L. (2019). Development of decreased-gluten wheat enabled by determination of the genetic basis of lys3a barley. *Plant Physiology*, *179*, 1692–1703

Oddy, J., Alarcón-Reverte, R., Wilkinson, M., Ravet, K., Raffan, S., Minter, A., Mead, A., Elmore, J.S., de Almeida, I.M., Cryer, N.C., Halford, N.G., & Pearce, S. (2021). Reduced free asparagine in wheat grain resulting from a natural deletion of TaASN-B2: investigating and exploiting diversity in the asparagine synthetase gene family to improve wheat quality. *BMC plant biology*, *21*. https://doi.org/10.1186/s12870-021-03058-7

Oddy, J., Raffan, S., Wilkinson, M.D., Elmore, J.S., & Halford, N.G. (2020). Stress, nutrients and genotype: understanding and managing asparagine accumulation in wheat grain. *CABI Agriculture and Bioscience*, *1*. https://doi.org/10.1186/s43170-020-00010-x

Oddy, J., Raffan, S., Wilkinson, M.D., Elmore, J.S., & Halford, N.G. (2022). Understanding the Relationships between Free Asparagine in Grain and Other Traits to Breed Low-Asparagine Wheat. *Plants*, *11*, 669

For Review Only

Pedersen, TL. (2021). ggraph: An Implementation of Grammar of Graphics for Graphs and Networks. R package version 2.0.5. https://CRAN.R-project.org/package=ggraph

Peng, Y., Liu, H., Chen, J., Shi, T., Zhang, C., Sun, D., He, Z., Hao, Y., & Chen, W. (2018). Genome-wide association studies of free amino acid levels by six multi-locus models in bread wheat. *Frontiers in Plant Science*, *9*, 1196

Prasanna, B.M., Palacios-Rojas, N., Hossain, F., Muthusamy, V., Menkir, A., Dhliwayo, T., Ndhlela, T., San Vicente, F., Nair, S.K., & Vivek, B.S. (2020). Molecular breeding for nutritionally enriched maize: status and prospects. *Frontiers in Genetics*, 1392

Przewieslik-Allen, A.M., Wilkinson, P.A., Burridge, A.J., Winfield, M.O., Dai, X., Beaumont, M., King, J., Yang, C., Griffiths, S., & Wingen, L.U. (2021). The role of gene flow and chromosomal instability in shaping the bread wheat genome. *Nature Plants*, *7*, 172–183

R Core Team (2021). R: A language and environment for statistical computing. R Foundation for Statistical Computing,Vienna, Austria. URL https://www.R-project.org/.

Raffan, S., Sparks, C., Huttly, A., Hyde, L., Martignago, D., Mead, A., Hanley, S.J., Wilkinson, P.A., Barker, G., Edwards, K.J., Curtis, T.Y., Usher, S., Kosik, O., & Halford, N.G. (2021). Wheat with greatly reduced accumulation of free asparagine in the grain, produced by CRISPR/Cas9 editing of asparagine synthetase gene TaASN2. *Plant Biotechnology Journal*, *19*. https://doi.org/10.1111/pbi.13573

Rapp, M., Schwadorf, K., Leiser, W.L., Würschum, T., & Longin, C.F.H. (2018). Assessing the variation and genetic architecture of asparagine content in wheat: What can plant

For Review Only

breeding contribute to a reduction in the acrylamide precursor? *Theoretical and Applied Genetics*, . https://doi.org/10.1007/s00122-018-3163-x

Rustgi, S., Shewry, P., Brouns, F., Deleu, L.J., & Delcour, J.A. (2019). Wheat seed proteins: factors influencing their content, composition, and technological properties, and strategies to reduce adverse reactions. *Comprehensive Reviews in Food Science and Food Safety*, *18*, 1751–1769

Shewry, P.R., Lovegrove, A., Wingen, L.U., & Griffiths, S. (2022). Opinion exploiting genomics to improve the benefits of wheat: Prospects and limitations. *Journal of Cereal Science*, *105*, 10344

Shorinola, O., Balcárková, B., Hyles, J., Tibbits, J.F.G., Hayden, M.J., Holušova, K., Valárik, M., Distelfeld, A., Torada, A., & Barrero, J.M. (2017). Haplotype analysis of the pre-harvest sprouting resistance locus Phs-A1 reveals a causal role of TaMKK3-A in global germplasm. *Frontiers in Plant Science*, *8*, 1555

Shorinola, O., Simmonds, J., Wingen, L.U., & Uauy, C. (2022). Trend, population structure, and trait mapping from 15 years of national varietal trials of UK winter wheat. *G3*, *12*, jkab415

Walkowiak, S., Gao, L., Monat, C., Haberer, G., Kassa, M.T., Brinton, J., Ramirez-Gonzalez, R.H., Kolodziej, M.C., Delorean, E., Thambugala, D., Klymiuk, V., Byrns, B., Gundlach, H., Bandi, V., Siri, J.N., Nilsen, K., Aquino, C., Himmelbach, A., Copetti, D., Ban, T., Venturini, L., Bevan, M., Clavijo, B., Koo, D.H., Ens, J., Wiebe, K., N'Diaye, A., Fritz, A.K., Gutwin, C., Fiebig, A., Fosker, C., Fu, B.X., Accinelli, G.G., Gardner, K.A., Fradgley, N., Gutierrez-Gonzalez, J., Halstead-Nussloch, G., Hatakeyama, M., Koh, C.S., Deek, J., Costamagna, A.C., Fobert, P., Heavens, D., Kanamori, H.,

For Review Only

Kawaura, K., Kobayashi, F., Krasileva, K., Kuo, T., McKenzie, N., Murata, K., Nabeka, Y., Paape, T., Padmarasu, S., Percival-Alwyn, L., Kagale, S., Scholz, U., Sese, J., Juliana, P., Singh, R., Shimizu-Inatsugi, R., Swarbreck, D., Cockram, J., Budak, H., Tameshige, T., Tanaka, T., Tsuji, H., Wright, J., Wu, J., Steuernagel, B., Small, I., Cloutier, S., Keeble-Gagnère, G., Muehlbauer, G., Tibbets, J., Nasuda, S., Melonek, J., Hucl, P.J., Sharpe, A.G., Clark, M., Legg, E., Bharti, A., Langridge, P., Hall, A., Uauy, C., Mascher, M., Krattinger, S.G., Handa, H., Shimizu, K.K., Distelfeld, A., Chalmers, K., Keller, B., Mayer, K.F.X., Poland, J., Stein, N., McCartney, C.A., Spannagl, M., Wicker, T., & Pozniak, C.J. (2020). Multiple wheat genomes reveal global variation in modern breeding. *Nature*, *588*. https://doi.org/10.1038/s41586-020-2961-x

Wang, L., Zhong, M., Li, X., Yuan, D., Xu, Y., Liu, H., He, Y., Luo, L., & Zhang, Q. (2008). The QTL controlling amino acid content in grains of rice (*Oryza sativa*) are co-localized with the regions involved in the amino acid metabolism pathway. *Molecular Breeding*, *21*, 127–137

Wei, T., and Simko, V. (2021). R package 'corrplot': Visualization of a Correlation Matrix (Version 0.92). Available from https://github.com/taiyun/corrplot

Wickham, H. (2011). The split-apply-combine strategy for data analysis. *Journal of Statistical Software*, *40*, 1–29

Wickham, H. (2016) ggplot2: Elegant Graphics for Data Analysis. Springer-Verlag New York.

Wickham et al., (2019). Welcome to the tidyverse. *Journal of Open Source Software*, 4(43), 1686, https://doi.org/10.21105/joss.01686

For Review Only

Wickham, H., François, R., Henry, L., and Müller, K. (2022). dplyr: A Grammar of Data Manipulation. R package version 1.0.8. https://CRAN.R-project.org/package=dplyr

Wilke, CO. (2020). cowplot: Streamlined Plot Theme and Plot Annotations for 'ggplot2'. R package version 1.1.1. https://CRAN.R-project.org/package=cowplot

Wilkinson, P.A., Allen, A.M., Tyrrell, S., Wingen, L.U., Bian, X., Winfield, M.O., Burridge, A., Shaw, D.S., Zaucha, J., Griffiths, S., Davey, R.P., Edwards, K.J., & Barker, G.L.A. (2020). CerealsDB - New tools for the analysis of the wheat genome: Update 2020. *Database*, *2020*. https://doi.org/10.1093/database/baaa060

Wilkinson, P.A., Winfield, M.O., Barker, G.L.A., Allen, A.M., Burridge, A., Coghill, J.A., & Edwards, K.J. (2012). CerealsDB 2.0: an integrated resource for plant breeders and scientists. *BMC Bioinformatics*, *13*, 1–6

Xie, Y., Malunga, L.N., Ames, N.P., Waterer, J., Khorshidi, A.S., & Scanlon, M.G. (2021). Effects of growing environment, genotype, and commercial fertilization levels on free asparagine concentration in Western Canadian wheat. *Cereal Chemistry*, *98*. https://doi.org/10.1002/cche.10364

Yoo, S.-C. (2017). Quantitative trait loci controlling the amino acid content in rice (*Oryza sativa* L.). *Journal of Plant Biotechnology*, *44*, 349–355

Zhong, M., Wang, L., Yuan, D., Luo, L., Xu, C., & He, Y. (2011). Identification of QTL affecting protein and amino acid contents in rice. *Rice Science*, *18*, 187–195

For Review Only

Figure Captions

Figure 1. Characterisation of the Robigus × Claire mapping population. a. Measurements of amino acids in the 2017–2018 (H18) and 2018–2019 (H19) harvest years. **b.** Principal component analysis of all traits in both years along the first two principal components. **c.** Correlation network analysis of all traits across both years (GH omitted, Kendall correlation, only links with significance <0.001 shown).

Figure 2. Relationships between free asparagine/lysine and other agronomic measurements. a. Linear modelling of free asparagine content against grain yield. The grey shaded ribbon shows 95% prediction intervals sampled from the posterior distribution. **b.** and **c.** Parameter values from multiple linear modelling of asparagine **(b.)** and lysine **(c.)** as explained by other traits measured in this population.

Figure 3. Identification of QTL controlling free asparagine and free lysine. a. Multi – environment genome scan plot for asparagine. **b.** Multi – environment genome scan plot for lysine. **c.** Impact of the asparagine QTL on free asparagine concentrations in both field trials. **d.** Impact of the lysine QTL on free lysine concentrations in both field trials. Error bars show plus and minus two times standard error of the mean. Significance values are taken from the corresponding years of the multi-environment linkage analysis.

Figure 4. Variation explained by heritability, genomic prediction, and QTL. a. Genomic prediction accuracy between years. **b.** Additive QTL effects and genomic prediction (rrBLUP) accuracy (yellow and red marks, respectively) plotted alongside broad-sense heritability

For Review Only

(shown as bars). Bars are shaded according to the trait group that they belong to (amino acid, size, or other).

Figure 5. Analysis of the *TaHDT-A1* candidate gene for lysine QTL between parents Claire and Robigus. a. Pairwise alignment of the two genes. **b.** Percentage identity calculated as a sliding window average of 100 bp. **c.** Expression of the four transcripts throughout development in variety Azhurnaya.

For Review Only

Table 1. Multi-environment QTL for measured amino acids. Chr. (Chromosome), cM (centimorgan), Mbp (megabase pair location in Chinese Spring v1.0).

Trait	Multi-environment single trait linkage analysis (H18 and H19)							
	Marker	Chr.	cM	Mbp	$-\log_{10}(p)$	H18 (%)	H19 (%)	High val.
Ala	WC.0223839	7B	211.2	719	5.03	7.1	5.7	Robigus
Asn	WC.0221262	4B	114.47	601	5.96	2.6	14.8	Robigus
Asp	WC.0218489	1B	54.4	530	5.4	8	5.9	Claire
	WC.0214359	3A2	2.3	738	7.95	7.3	15.3	Robigus
	WC.0221037	4A	148.8	703	8.08	12.6	9.3	Claire
	WC.0227146	4D	48.8	16	3.7	5.5	4.1	Claire
Gln	WC.0221302	4B	103.7	547	3.5	5.4	4.5	Robigus
	WC.0228471	6B	19.7	25	5.09	8.2	6.7	Claire
Glu	WC.0221329	4B	100.8	518	4.27	3.7	10.1	Robigus
Gly	WC.0226796	4B	155.2	327	4.26	3.2	5.3	Robigus
Iso	WC.0223785	7B	211.2	717	3.6	6.8	3.7	Robigus
Lys	WC.0218011	1A2	27.3	593	4.95	12.1	2.6	Claire
Phe	WC.0220622	3B1	78.1	116	3.83	6.2	5.6	Robigus

For Review Only

Table 2. Multi-environment QTL impacting both amino acids and other traits on chromosomes 4A, 4B, and 4D. Chr. (Chromosome), cM (centimorgan), Mbp (megabase pair location in Chinese Spring v1.0).

Chr.	Multi-environment single trait linkage analysis (H18 and H19)							
	**Trait**	**Marker**	**cM**	**Mbp**	**-log$_{10}$(*p*)**	**H18 (%)**	**H19 (%)**	**High val.**
4A	Asp	WC.0221037	148.8	703	8.08	12.6	9.3	Claire
	KHI	WC.0221037	148.8	703	8.26	14.8	14.7	Robigus
	HFN	WC.0188904	147.1	733	8.24	11.5	10.3	Robigus
	Area	WC.0220938	149.7	709	2.22	3	3.2	Claire
	Length	WC.0221119	149.7	702	7.12	1.8	6.5	Claire
4B	Asn	WC.0221262	114.47	601	5.96	2.6	14.8	Robigus
	KHI	WC.0226741	110.8	594	4.30	4.2	8.6	Robigus
	Diam	WC.0226868	82.5	32	18.53	17.7	18.7	Claire
	Height	WC.0226868	82.5	32	17.98	19.8	20.7	Claire
	Weight	WC.0226868	82.5	32	9.25	12.2	14.1	Claire
4D	Asp	WC.0227146	48.8	16	3.7	5.5	4.1	Claire
	Width	WC.0227146	48.8	16	5.98	8	8.9	Robigus
	Diam	WC.0227146	48.8	16	7.84	7.6	8.1	Robigus
	HFN	WC.0227149	56.9	17	10.92	23.5	5.6	Robigus
	Height	WC.0213051	56.9	17	28.97	27.8	38.9	Robigus

For Review Only

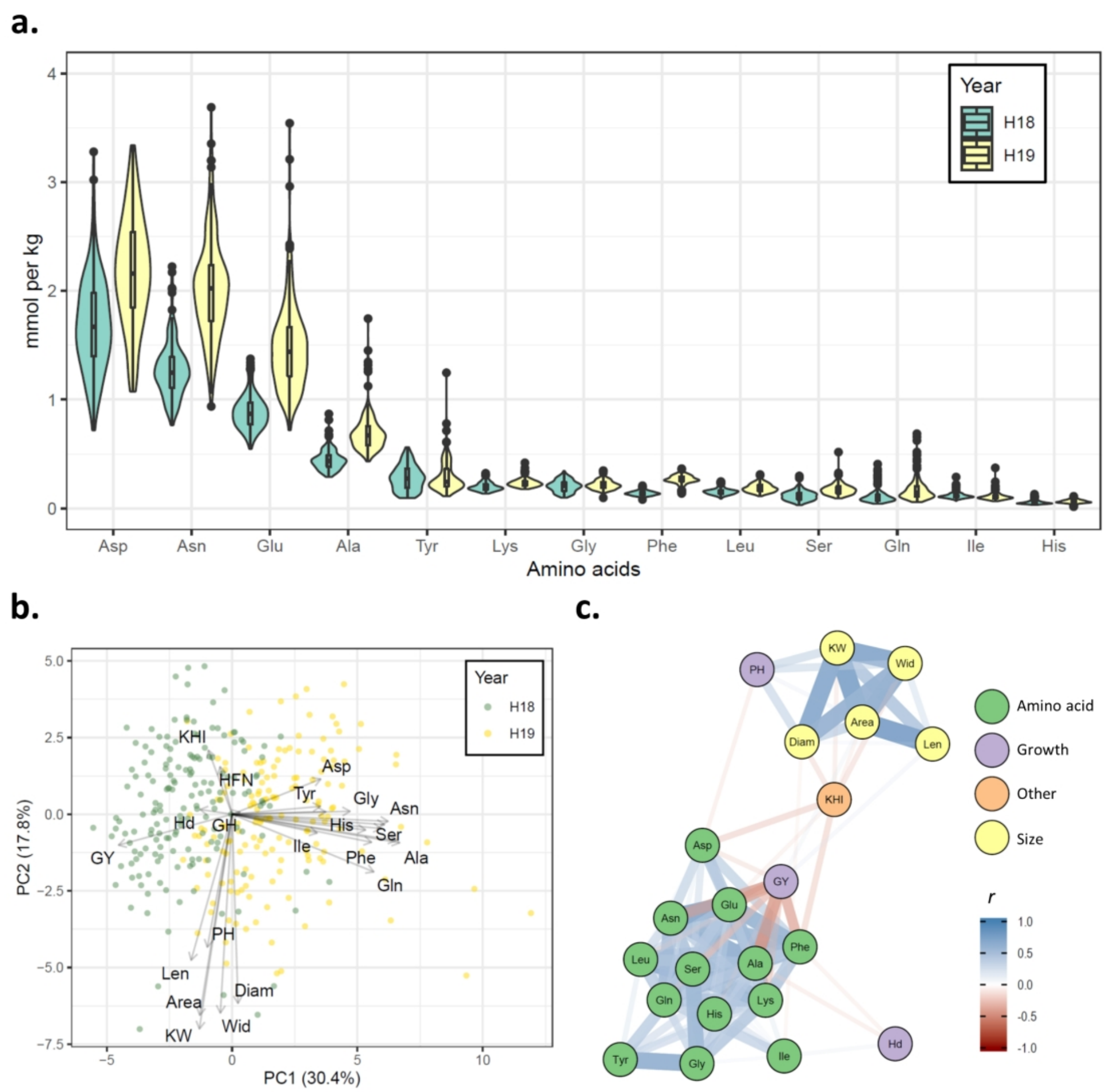

Figure 1. Characterisation of the Robigus × Claire mapping population. a. Measurements of amino acids in the 2017–2018 (H18) and 2018–2019 (H19) harvest years. b. Principal component analysis of all traits in both years along the first two principal components. c. Correlation network analysis of all traits across both years (GH omitted, Kendall correlation, only links with significance <0.001 shown).

190x187mm (300 x 300 DPI)

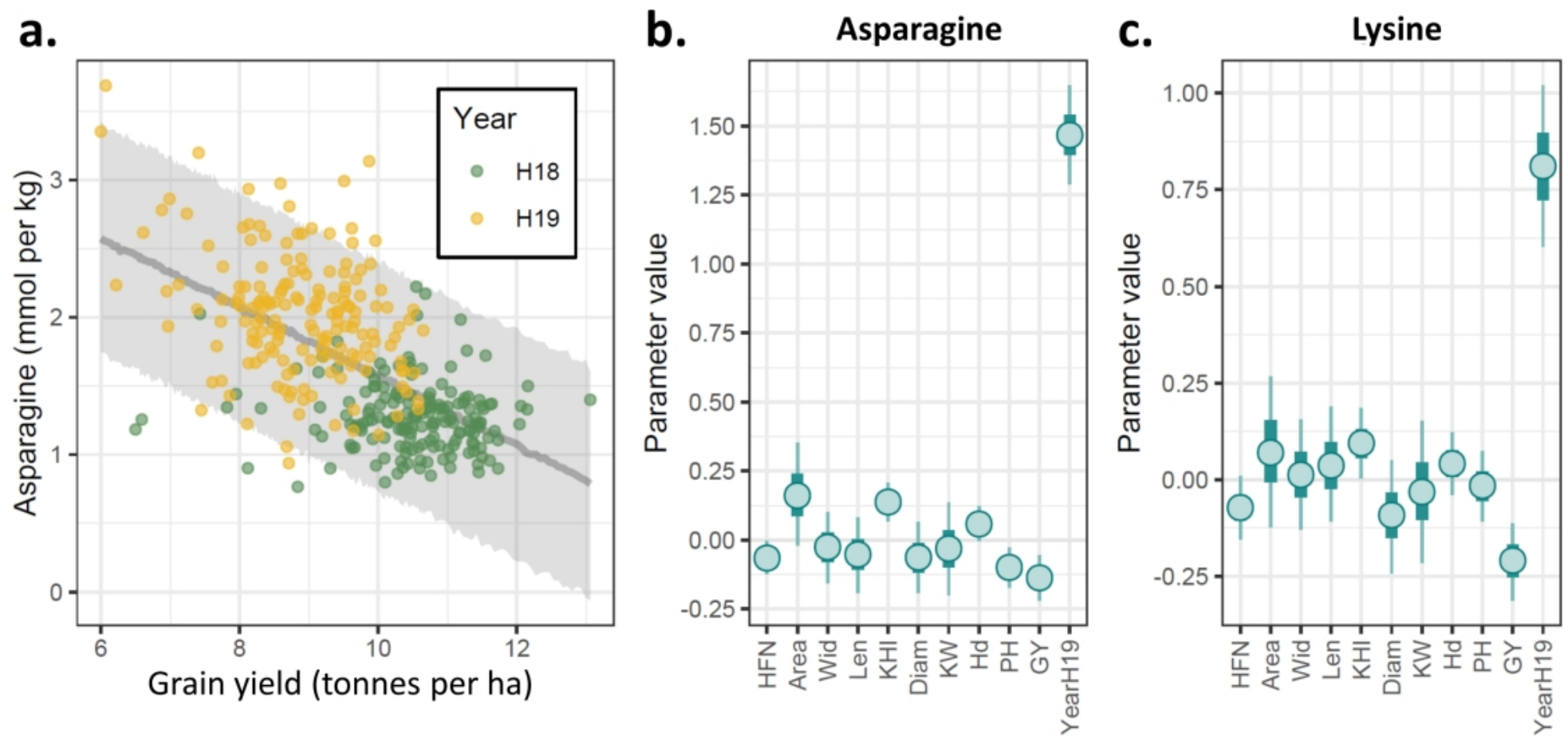

Figure 2. Relationships between free asparagine/lysine and other agronomic measurements. a. Linear modelling of free asparagine content against grain yield. The grey shaded ribbon shows 95% prediction intervals sampled from the posterior distribution. b. and c. Parameter values from multiple linear modelling of asparagine (b.) and lysine (c.) as explained by other traits measured in this population.

252x119mm (300 x 300 DPI)

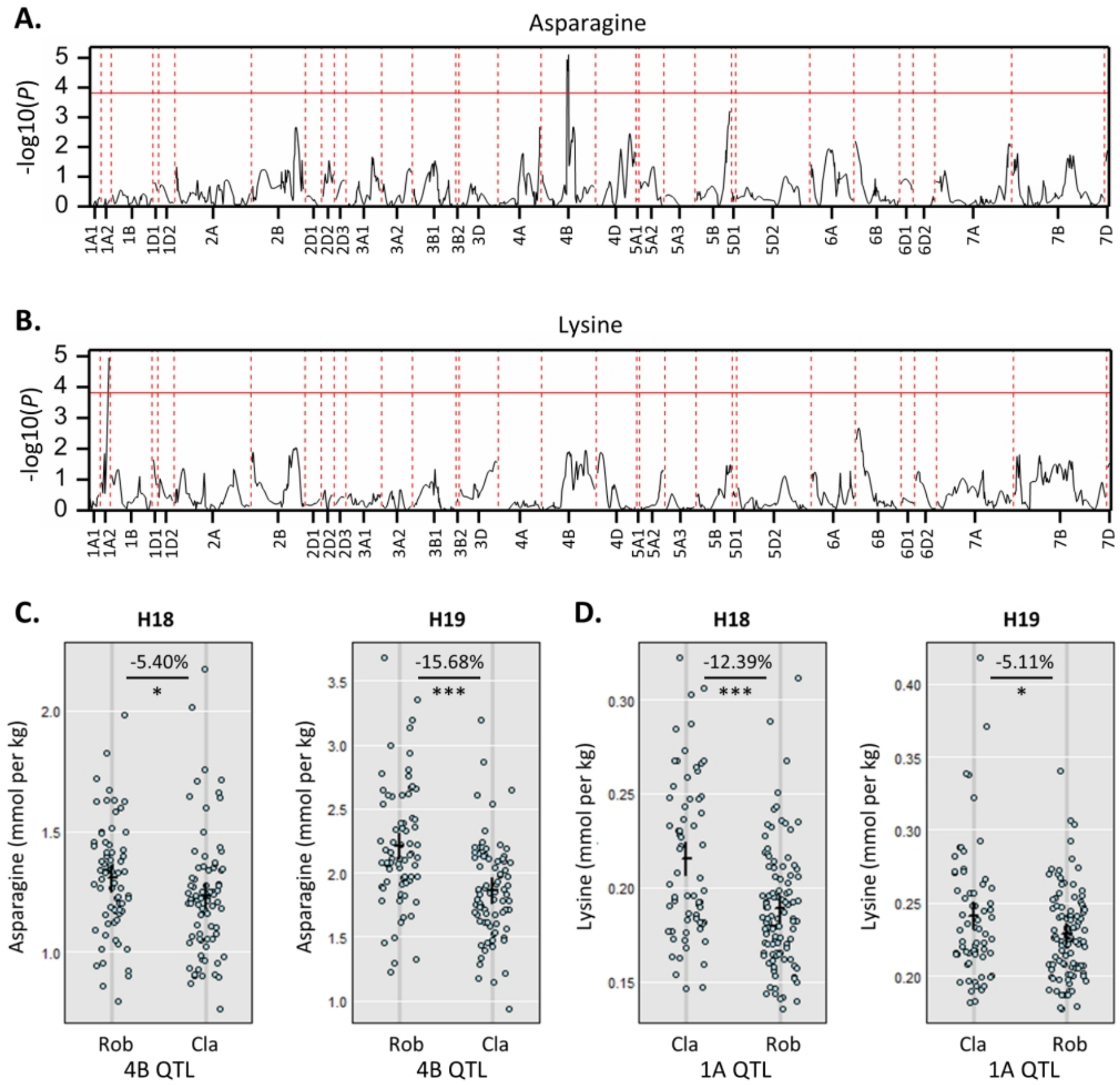

Figure 3. Identification of QTL controlling free asparagine and free lysine. a. Multi – environment genome scan plot for asparagine. b. Multi – environment genome scan plot for lysine. c. Impact of the asparagine QTL on free asparagine concentrations in both field trials. d. Impact of the lysine QTL on free lysine concentrations in both field trials. Error bars show plus and minus two times standard error of the mean. Significance values are taken from the corresponding years of the multi-environment linkage analysis.

190x175mm (300 x 300 DPI)

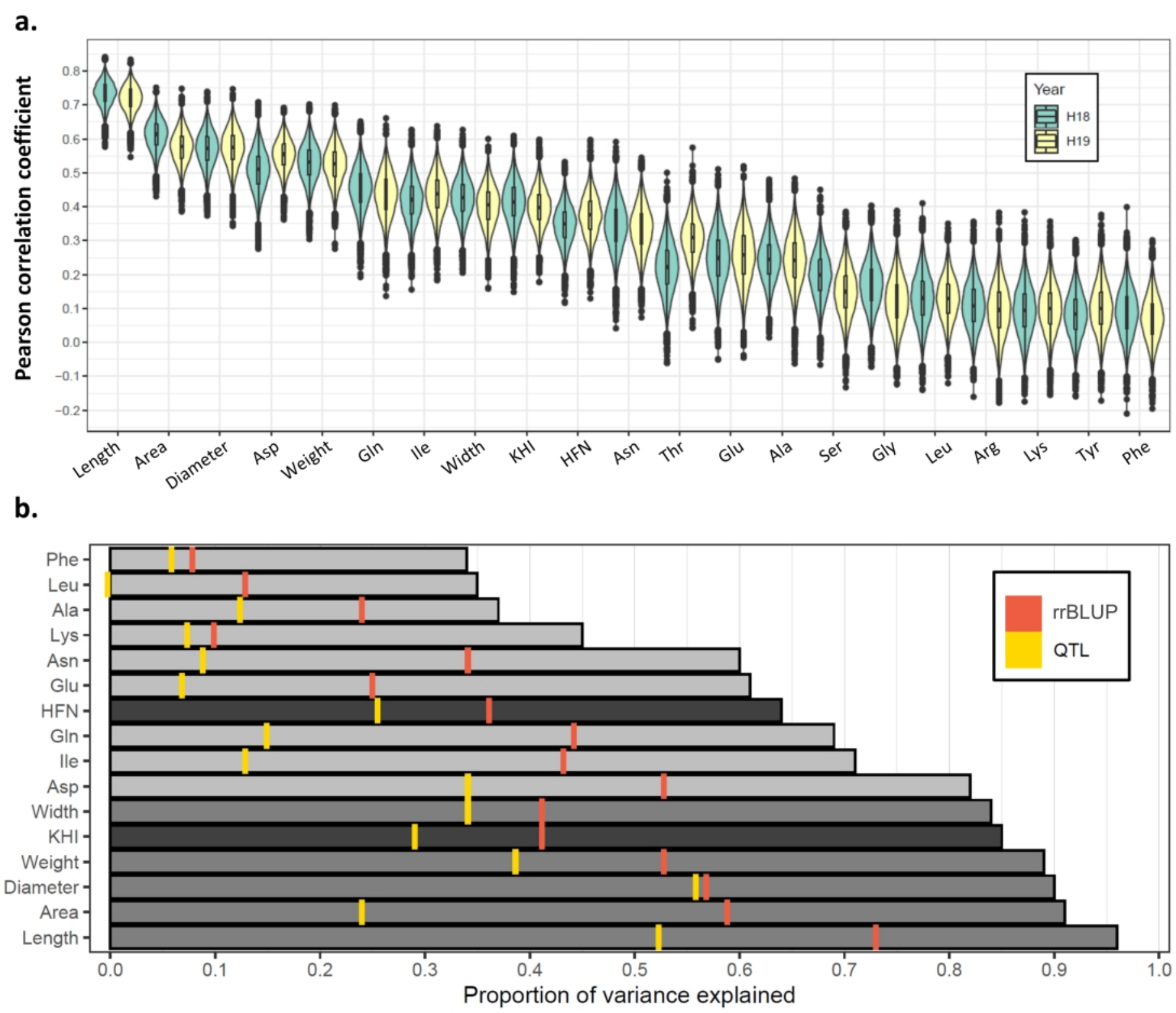

Figure 4. Variation explained by heritability, genomic prediction, and QTL. a. Genomic prediction accuracy between years. b. Additive QTL effects and genomic prediction (rrBLUP) accuracy (yellow and red marks, respectively) plotted alongside broad-sense heritability (shown as bars). Bars are shaded according to the trait group that they belong to (amino acid, size, or other).

252x209mm (300 x 300 DPI)

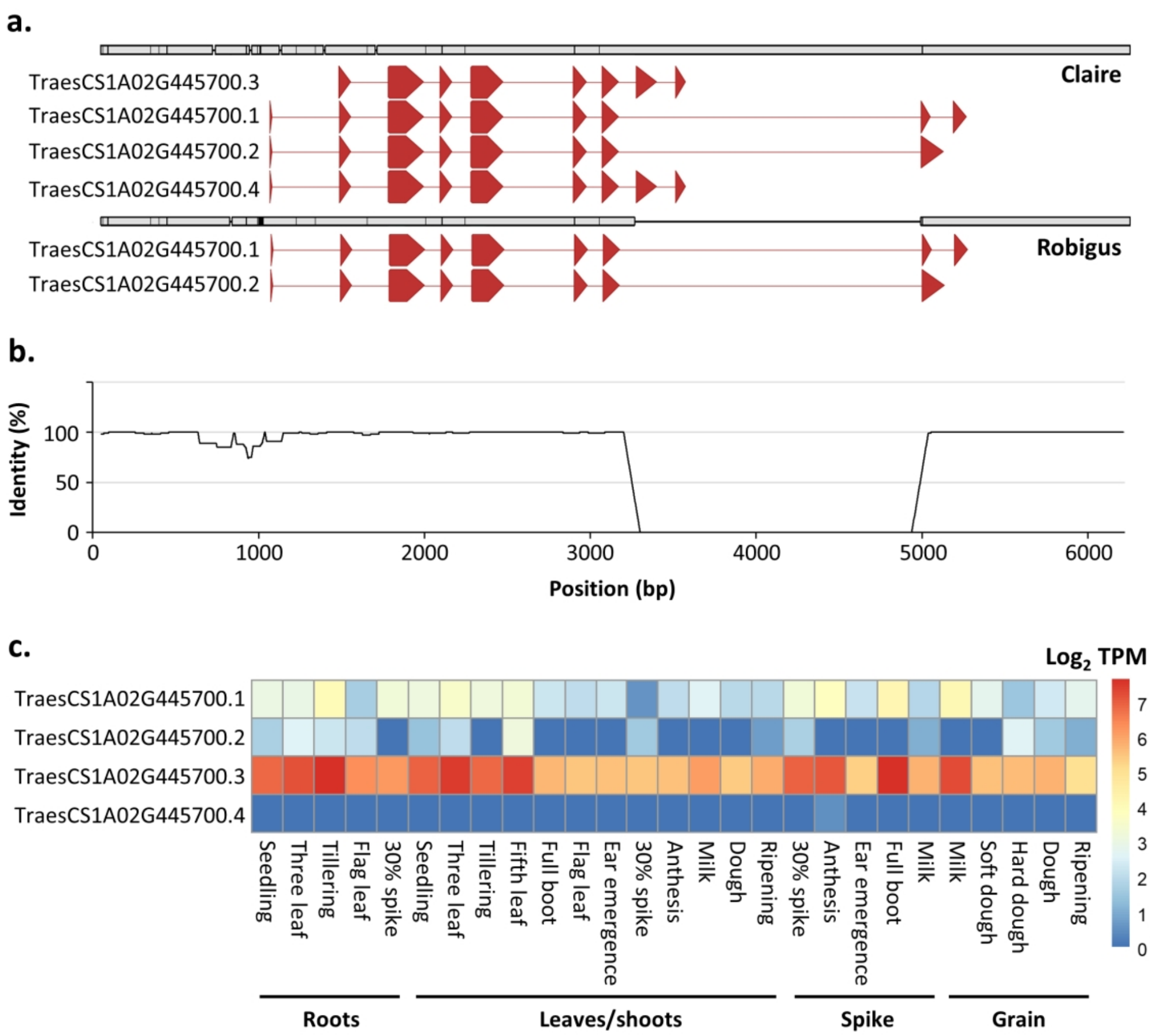

Figure 5. Analysis of the TaHDT-A1 candidate gene for lysine QTL between parents Claire and Robigus. a. Pairwise alignment of the two genes. b. Percentage identity calculated as a sliding window average of 100 bp. c. Expression of the four transcripts throughout development in variety Azhurnaya.

190x170mm (300 x 300 DPI)

Genetic control of grain amino acid composition in a UK soft wheat mapping population

Joseph Oddy[1], Monika Chhetry[2], Rajani Awal[2], John Addy[1], Mark Wilkinson[1], Dan Smith[1], Robert King[1], Chris Hall[1], Rebecca Testa[1], Eve Murray[1], Sarah Raffan[1], Tanya Y. Curtis[3], Luzie Wingen[2], Simon Griffiths[2], Simon Berry[4], J. Stephen Elmore[5], Nicholas Cryer[6], Isabel Moreira de Almeida[7], and Nigel G. Halford[1*]

[1]*Rothamsted Research, Harpenden, Hertfordshire AL5 2JQ, UK*

[2]*John Innes Centre, Norwich Research Park, Colney Lane, Norwich, UK*

[3]*Curtis Analytics Limited, Discovery Park, Sandwich, UK*

[4]*Limagrain UK Ltd, Market Rasen LN7 6DT, UK*

[5]*Department of Food & Nutritional Sciences, University of Reading, RG6 6DZ, UK*

[6]*Mondelēz UK R&D Ltd, Bournville Lane, Bournville, Birmingham, B30 2LU, UK*

[7]*Mondelēz R&D International, 6 Rue René Razel, 91400 Saclay, France*

For Review Only

Supplementary figures

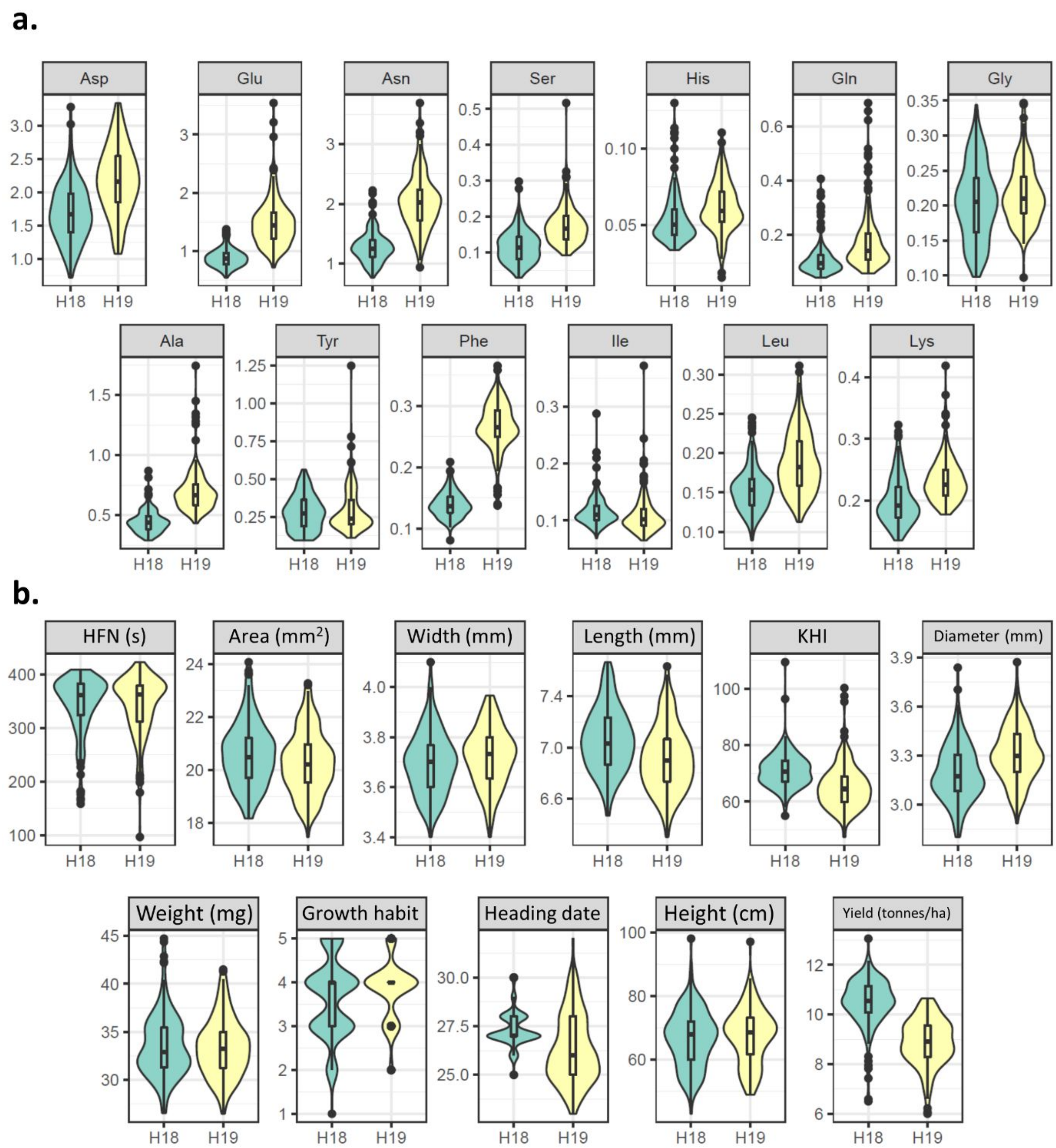

Supplementary figure 1. Trait measurements from the Claire x Robigus mapping population grown across two years (H18 and H19). a. Concentration of amino acids (mmol per kg) in wheat grain in both environments. **b.** Measurements of other quality and agronomic traits across both environments.

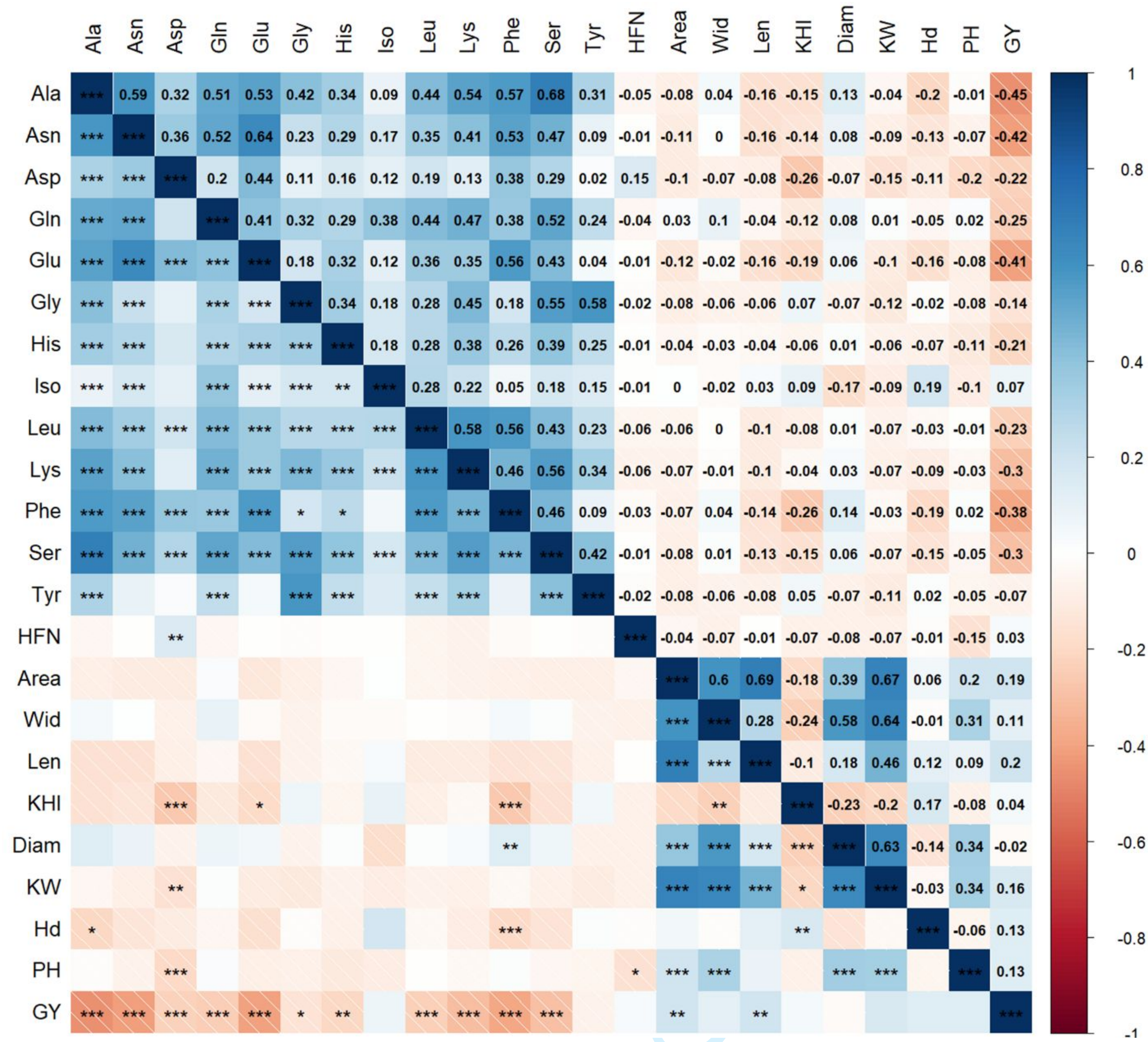

Supplementary figure 2. Correlation of traits taken from both H18 and H19 environments. Kendall correlation coefficients shown in upper right triangle and significance asterisks from adjusted p values (Bonferroni correction) shown in lower left triangle. HFN (Hagberg falling number), KHI (kernel hardness index), Diam (diameter), KW (kernel weight), Hd (heading date), PH (plant height), GY (grain yield).

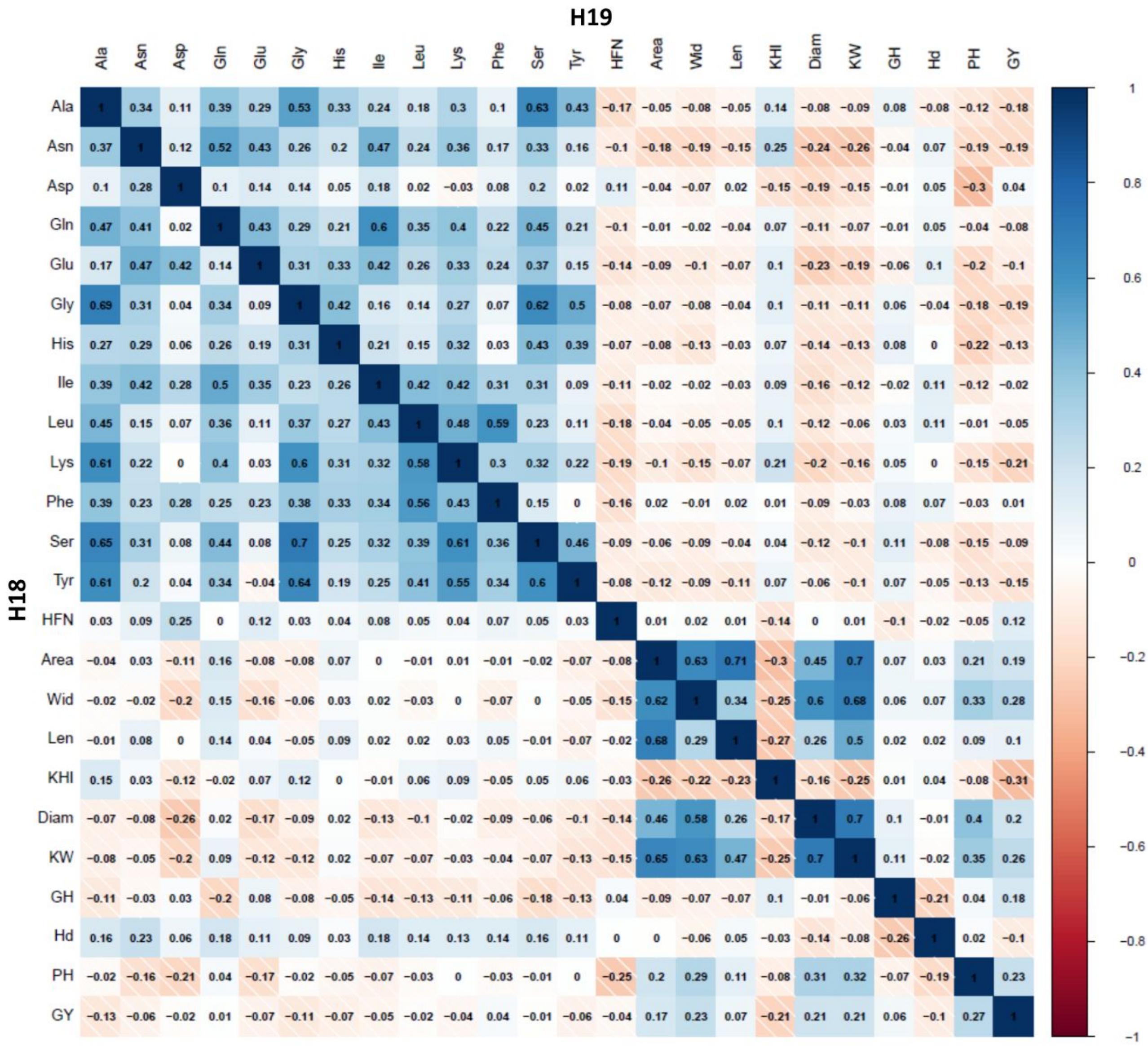

Supplementary figure 3. Kendall correlation coefficients between traits within each environment. H18 is the bottom left triangle, H19 is the upper right triangle.

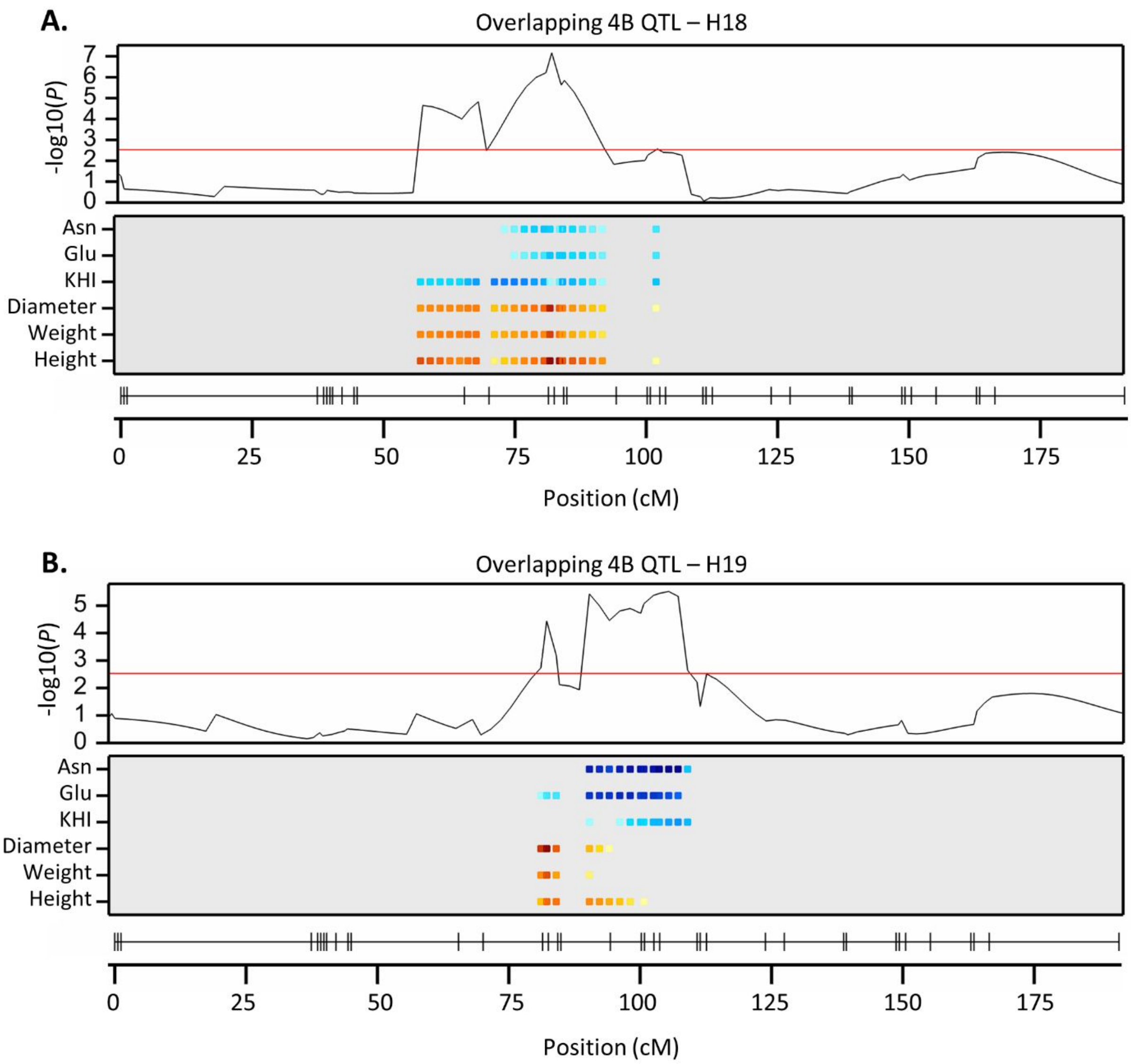

Supplementary figure 4. Multi-trait analysis of the asparagine QTL on 4B in the Claire x Robigus mapping population. Blue indicates Robigus additive allele whilst red indicates Claire additive allele. The darkness of colour corresponds to the magnitude of the effect.

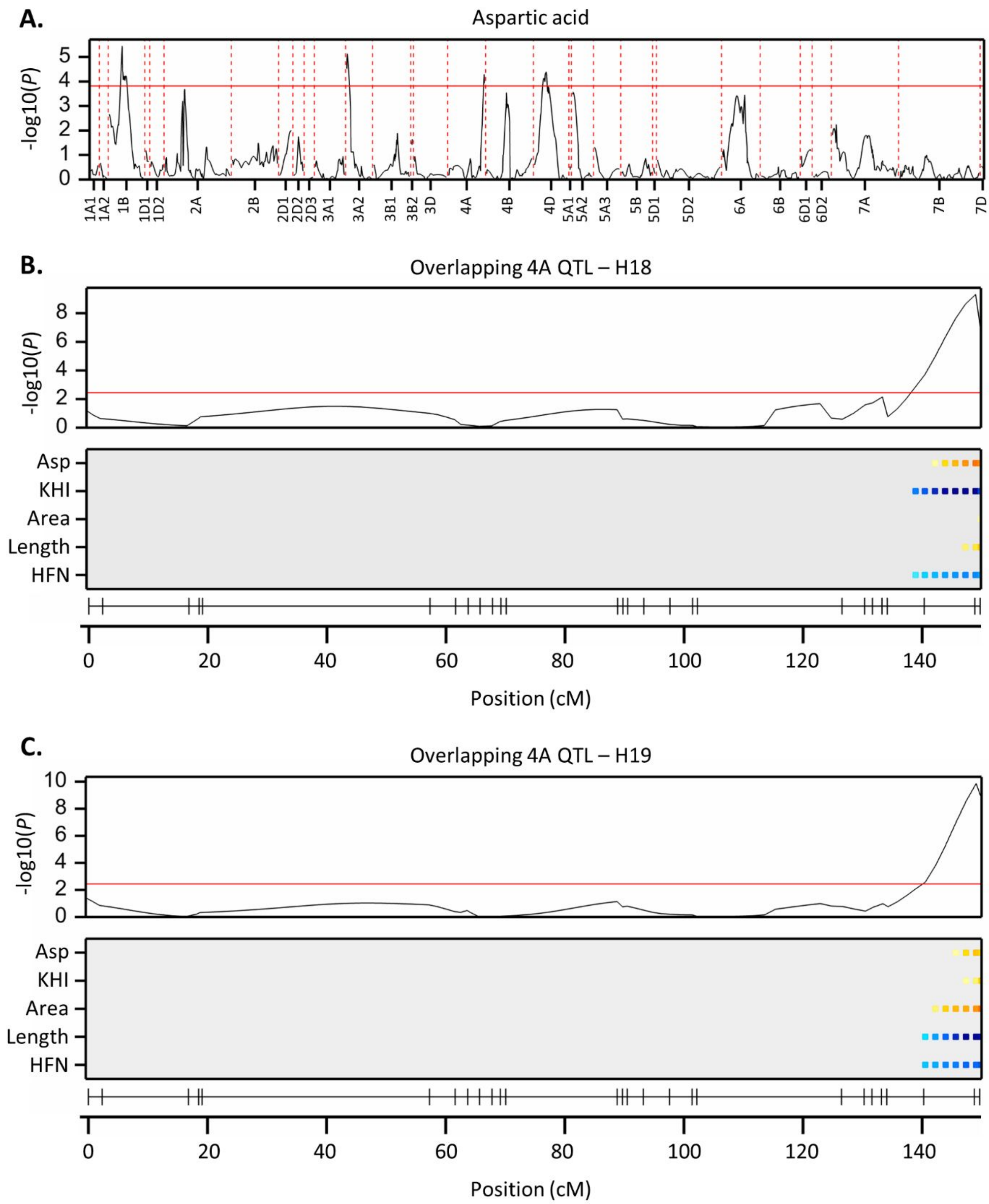

Supplementary figure 5. Multi-environment and multi-trait linkage analysis of aspartic acid QTL in the Claire x Robigus mapping population.

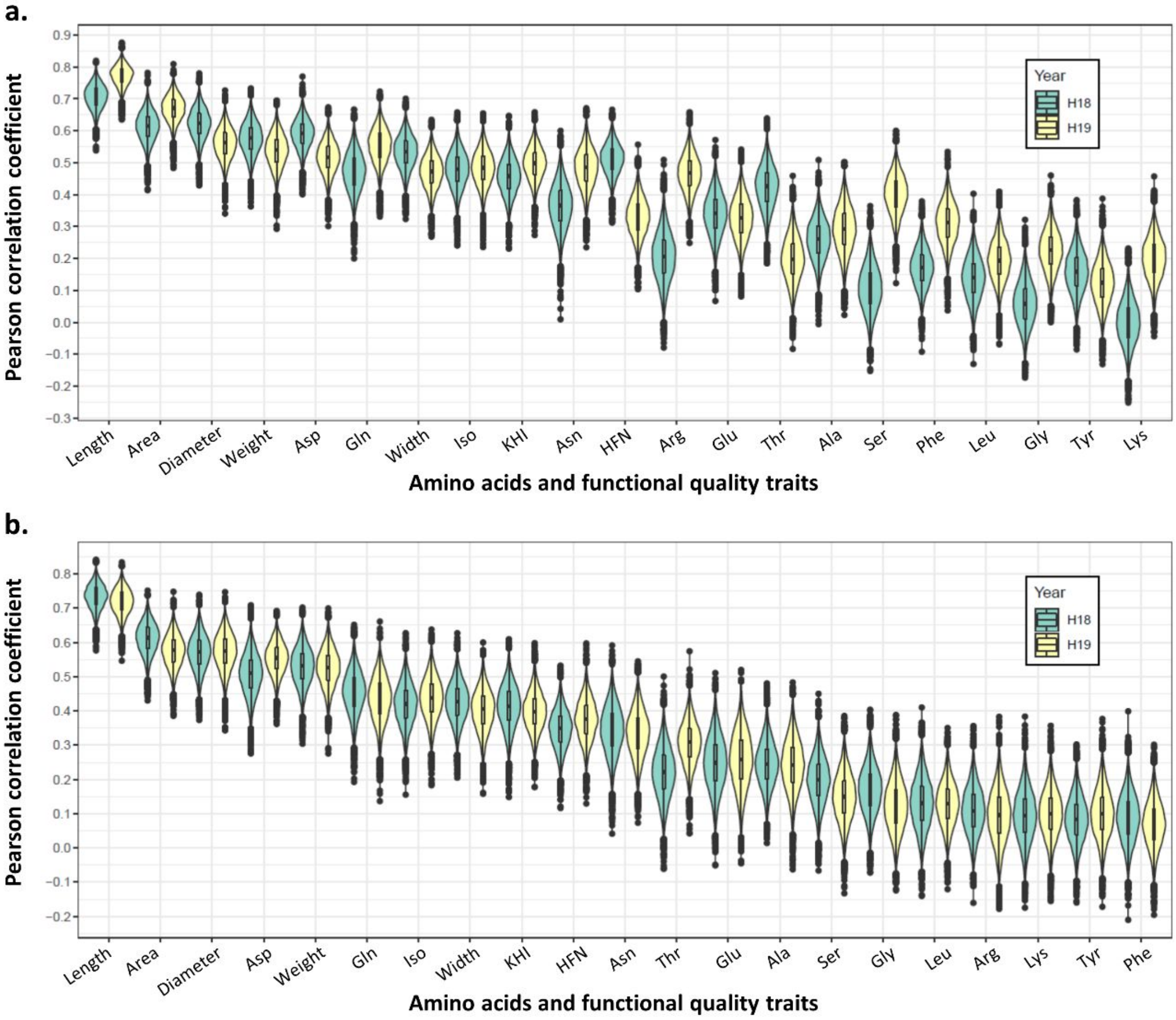

Supplementary figure 6. Accuracy of genomic selection for each trait measured in the mapping population using within (a.) and between (b.) year prediction.

Only

Genetic control of grain amino acid composition in a UK soft wheat mapping population

Joseph Oddy[1], Monika Chhetry[2], Rajani Awal[2], John Addy[1], Mark Wilkinson[1], Dan Smith[1], Robert King[1], Chris Hall[1], Rebecca Testa[1], Eve Murray[1], Sarah Raffan[1], Tanya Y. Curtis[3], Luzie Wingen[2], Simon Griffiths[2], Simon Berry[4], J. Stephen Elmore[5], Nicholas Cryer[6], Isabel Moreira de Almeida[7], and Nigel G. Halford[1*]

[1]*Rothamsted Research, Harpenden, Hertfordshire AL5 2JQ, UK*

[2]*John Innes Centre, Norwich Research Park, Colney Lane, Norwich, UK*

[3]*Curtis Analytics Limited, Discovery Park, Sandwich, UK*

[4]*Limagrain UK Ltd, Market Rasen LN7 6DT, UK*

[5]*Department of Food & Nutritional Sciences, University of Reading, RG6 6DZ, UK*

[6]*Mondelēz UK R&D Ltd, Bournville Lane, Bournville, Birmingham, B30 2LU, UK*

[7]*Mondelēz R&D International, 6 Rue René Razel, 91400 Saclay, France*

For Review Only

Supplementary tables

Supplementary table 1. Broad sense heritability estimates, Kendall correlation coefficients for traits across both environments, and within and between environments average genomic prediction accuracies (as Pearson correlation coefficients). Heritability estimates and genomic prediction performed on transformed data, Kendall correlation coefficients performed on non-transformed data. KHI (Kernel Hardness Index), HFN (Hagberg Falling Number), h^2 (heritability), r (Kendall correlation coefficient), GP (genomic prediction), H18 (first field trial), H19 (second field trial).

			Within environment GP *r*			Between environments GP *r*		
**Traits**	h^2	r	**H18**	**H19**	**Mean**	**H18 train**	**H19 train**	**Mean**
**Amino acids**								
Alanine	0.37	0.00	0.26	0.29	0.27	0.24	0.24	0.24
Asparagine	0.60	0.34	0.36	0.48	0.42	0.35	0.33	0.34
Aspartic acid	0.82	0.51	0.59	0.52	0.55	0.51	0.55	0.53
Glutamic acid	0.61	0.33	0.34	0.33	0.33	0.25	0.26	0.25
Glutamine	0.69	0.30	0.47	0.55	0.51	0.45	0.43	0.44
Glycine	0.00	-0.12	0.06	0.22	0.14	0.17	0.12	0.15
Isoleucine	0.71	0.39	0.48	0.48	0.48	0.42	0.44	0.43
Leucine	0.35	0.18	0.14	0.19	0.17	0.13	0.13	0.13
Lysine	0.45	0.15	0.00	0.20	0.10	0.09	0.10	0.10
Phenylalanine	0.34	0.17	0.17	0.31	0.24	0.09	0.07	0.08
Serine	0.00	-0.14	0.11	0.40	0.25	0.20	0.15	0.17
Tyrosine	0.00	-0.23	0.16	0.12	0.14	0.08	0.10	0.09
**Functional traits**								
Area	0.91	0.63	0.61	0.67	0.64	0.61	0.58	0.59
Length	0.96	0.77	0.71	0.77	0.74	0.73	0.72	0.73
Width	0.84	0.55	0.53	0.47	0.50	0.43	0.40	0.41
Diameter	0.90	0.60	0.62	0.56	0.59	0.57	0.57	0.57
KHI	0.85	0.58	0.46	0.50	0.48	0.41	0.40	0.41
Weight	0.89	0.59	0.58	0.54	0.56	0.53	0.53	0.53
HFN	0.64	0.34	0.51	0.33	0.42	0.35	0.37	0.36

For Review Only

Supplementary table 2. Multi-environment QTL for measured amino acids. Chr (chromosome), cM (centimorgan), Mbp (megabase pair location in Chinese Spring), QTL x E (QTL by environment interaction), ratio (ratio of effects between years), S.E. (standard error of the mean), % Expl. (percentage of variance explained).

	Single trait linkage analysis (H18 and H19)							H18					H19				
Trait	Marker	Chr.	cM	Mbp	$-log_{10}(p)$	QTL x E	Ratio	Effect	S.E.	High value	*p*	% Expl.	Effect	S.E.	High value	*p*	% Expl.
Asparagine	WC.0221262	4B	114.47	601	5.96	yes	4.34	0.041	0.020	Robigus	0.040	2.6	0.178	0.034	Robigus	<0.001	14.8
Log_e alanine	WC.0218011	1A2	27.3	593	5.10	yes	0.34	0.065	0.016	Claire	<0.001	10.9	0.022	0.015	Robigus	0.159	1
	WC.0223839	7B	211.2	719	5.03	no	1.00	0.052	0.012	Robigus	<0.001	7.1	0.052	0.012	Robigus	<0.001	5.7
Aspartic acid	WC.0218489	1B	54.4	530	5.40	no	1.00	0.123	0.027	Claire	<0.001	8	0.123	0.027	Claire	<0.001	5.9
	WC.0214359	3A2	2.3	738	7.95	yes	1.68	0.118	0.029	Robigus	<0.001	7.3	0.198	0.033	Robigus	<0.001	15.3
	WC.0221037	4A	148.8	703	8.08	no	1.00	0.154	0.027	Claire	<0.001	12.6	0.154	0.027	Claire	<0.001	9.3
	WC.0227146	4D	48.8	16	3.70	no	1.00	0.102	0.028	Claire	<0.001	5.5	0.102	0.028	Claire	<0.001	4.1
Log_e glutamate	WC.0221329	4B	100.8	518	4.27	yes	2.41	0.034	0.013	Robigus	0.011	3.7	0.082	0.019	Robigus	<0.001	10.1
Log_e glutamine	WC.0218486	1B	117.93	660	2.16	yes	9.91	0.011	0.038	Robigus	0.760	0.1	0.109	0.038	Robigus	0.004	4.9
	WC.0221302	4B	103.7	547	3.50	no	1.00	0.105	0.029	Robigus	<0.001	5.4	0.105	0.029	Robigus	<0.001	4.5
	WC.0228471	6B	19.7	25	5.09	no	1.00	0.129	0.029	Claire	<0.001	8.2	0.129	0.029	Claire	<0.001	6.7
Glycine	WC.0218011	1A2	27.3	593	5.37	yes	0.53	0.017	0.004	Claire	<0.001	9.6	0.009	0.003	Robigus	0.005	4.3
	WC.0226796	4B	155.2	327	4.26	no	1.00	0.010	0.002	Robigus	<0.001	3.2	0.010	0.002	Robigus	<0.001	5.3
-1/Isoleucine	WC.0221386	4B	94.3	172	7.31	yes	5.10	0.161	0.120	Robigus	0.180	1	0.821	0.151	Robigus	<0.001	14.1
	WC.0223785	7B	211.2	717	3.60	no	1.00	0.421	0.115	Robigus	<0.001	6.8	0.421	0.115	Robigus	<0.001	3.7
Log_e lysine	WC.0218011	1A2	27.3	593	4.95	yes	0.38	0.064	0.014	Claire	<0.001	12.1	0.024	0.011	Claire	0.038	2.6
Phenylalanine	WC.0220622	3B1	78.1	116	3.83	yes	1.80	0.005	0.002	Robigus	0.001	6.2	0.009	0.003	Robigus	0.002	5.6
Log_e serine	WC.0226730	4B	123.8	632	3.39	yes	2.33	0.033	0.031	Claire	0.279	0.7	0.077	0.020	Robigus	<0.001	7.9

For Review Only

Supplementary table 3. Multi-environment QTL for selected grain traits. Chr (chromosome), cM (centimorgan), Mbp (megabase pair location in Chinese Spring), QTL x E (QTL by environment interaction), ratio (ratio of effects between years), S.E. (standard error of the mean), % Expl. (percentage of variance explained), KHI (Kernel Hardness Index), HFN (Hagberg Falling Number).

Trait	Single trait linkage analysis (H18 and H19)							H18					H19				
	Marker	Chr.	cM	Mbp	$-\log_{10}(p)$	QTLxE	Ratio	Effect	High value	S.E.	*p*	% Expl.	Effect	High value	S.E.	*p*	% Expl.
Log_e KHI	WC.0221037	4A	148.8	703	8.26	yes	1.41	0.032	Robigus	0.006	<0.001	14.8	0.045	Robigus	0.008	<0.001	14.7
	WC.0226741	4B	110.8	594	4.30	yes	2.06	0.017	Robigus	0.006	0.002	4.2	0.035	Robigus	0.008	<0.001	8.6
	WC.0222754	6A	83.22	108	6.09	yes	4.40	0.005	Claire	0.006	0.377	0.4	0.022	Robigus	0.008	0.007	3.4
	WC.0228678	7A	152.17	539	4.45	no	1.00	0.024	Claire	0.006	<0.001	8.1	0.024	Claire	0.006	<0.001	4.1
Area	WC.0217441	2A	70.8	176	7.48	yes	1.43	0.325	Robigus	0.080	<0.001	7.4	0.464	Robigus	0.080	<0.001	15.8
	WC.0220938	4A	149.7	709	2.22	no	1.00	0.208	Claire	0.076	0.006	3	0.208	Claire	0.076	0.006	3.2
	WC.0193228	7A	158.09	610	4.94	no	1.00	0.360	Robigus	0.082	<0.001	9.1	0.360	Robigus	0.082	<0.001	9.5
Length	WC.0225130	2A	67.2	166	8.15	no	1.00	0.077	Robigus	0.013	<0.001	9.6	0.077	Robigus	0.013	<0.001	9.1
	WC.0212864	3A1	92.1	63	4.86	no	1.00	0.058	Robigus	0.013	<0.001	5.5	0.058	Robigus	0.013	<0.001	5.1
	WC.0225952	3B1	128.8	739	7.18	no	1.00	0.072	Claire	0.013	<0.001	8.5	0.072	Claire	0.013	<0.001	8
	WC.0221119	4A	149.7	702	7.12	yes	1.91	0.034	Claire	0.014	0.019	1.8	0.065	Claire	0.014	<0.001	6.5
	WC.0221932	5A1	0.6	1	9.23	no	1.00	0.084	Robigus	0.014	<0.001	11.4	0.084	Robigus	0.014	<0.001	10.7
	WC.0223413	7A	171.6	641	7.23	no	1.00	0.072	Robigus	0.013	<0.001	8.4	0.072	Robigus	0.013	<0.001	7.9
	WC.0222406	7B	40.6	34	7.35	yes	1.54	0.048	Claire	0.014	0.001	3.7	0.074	Claire	0.014	<0.001	8.4
Width	WC.0217441	2A	72.25	176	4.88	no	1.00	0.031	Robigus	0.007	<0.001	6.1	0.031	Robigus	0.007	<0.001	6.9
	WC.0095497	2B	120.78	545	4.04	yes	3.44	0.009	Claire	0.008	0.257	0.5	0.031	Claire	0.008	<0.001	7.1
	WC.0227146	4D	48.8	16	5.98	no	1.00	0.035	Robigus	0.007	<0.001	8	0.035	Robigus	0.007	<0.001	8.9
	WC.0228194	6A	86.1	127	7.35	yes	0.59	0.044	Robigus	0.008	<0.001	12.9	0.026	Robigus	0.008	0.001	4.9
	WC.0228849	7A	174.8	641	3.04	no	1.00	0.024	Robigus	0.007	0.001	3.8	0.024	Robigus	0.007	0.001	4.2
	WC.0212528	7B	107.2	641	4.60	yes	0.11	0.028	Robigus	0.008	<0.001	5.2	0.003	Robigus	0.008	0.732	0.1
Diameter	WC.0217441	2A	72.25	176	3.62	no	1.00	0.030	Robigus	0.008	<0.001	3.1	0.030	Robigus	0.008	<0.001	3.2
	WC.0226154	3B1	81.6	237	9.72	no	1.00	0.051	Claire	0.008	<0.001	8.9	0.051	Claire	0.008	<0.001	9.4
	WC.0226868	4B	82.5	32	18.53	no	1.00	0.072	Claire	0.008	<0.001	17.7	0.072	Claire	0.008	<0.001	18.7
	WC.0227146	4D	48.8	16	7.84	no	1.00	0.047	Robigus	0.008	<0.001	7.6	0.047	Robigus	0.008	<0.001	8.1
	WC.0221859	5A2	2.3	30	5.62	no	1.00	0.038	Claire	0.008	<0.001	5	0.038	Claire	0.008	<0.001	5.2
	WC.0212957	6A	99.37	499	8.73	no	1.00	0.050	Robigus	0.008	<0.001	8.4	0.050	Robigus	0.008	<0.001	8.8
	WC.0222406	7B	40.6	34	4.43	no	1.00	0.034	Claire	0.008	<0.001	3.9	0.034	Claire	0.008	<0.001	4.1
Weight	WC.0217441	2A	72.25	176	6.45	no	1.00	0.936	Robigus	0.184	<0.001	8.4	0.936	Robigus	0.184	<0.001	9.7
	WC.0229571	2B	9.37	12	3.18	no	1.00	0.629	Claire	0.185	0.001	3.8	0.629	Claire	0.185	0.001	4.4
	WC.0226868	4B	82.5	32	9.25	no	1.00	1.127	Claire	0.182	<0.001	12.2	1.127	Claire	0.182	<0.001	14.1
	WC.0212957	6A	99.37	499	8.28	no	1.00	1.085	Robigus	0.186	<0.001	11.3	1.085	Robigus	0.186	<0.001	13.1
-1/(500-HFN)	WC.0188904	4A	147.1	733	8.24	no	1.00	0.001	Robigus	0.000	<0.001	11.5	0.001	Robigus	0.000	<0.001	10.3
	WC.0227149	4D	56.9	17	10.92	yes	0.00	0.001	Robigus	0.000	<0.001	23.5	0.000	Robigus	0.000	<0.001	5.6
Heading date	WC.0226616	4A	130.3	632	4.14	no	1.00	0.258	Claire	0.065	<0.001	8.6	0.258	Claire	0.065	<0.001	2.2
	WC.0221774	5A3	104.9	706	4.51	yes	3.88	0.146	Claire	0.065	0.024	2.8	0.566	Claire	0.124	<0.001	10.5
Plant height	WC.0226868	4B	82.5	32	17.98	no	1.00	3.921	Claire	0.444	<0.001	19.8	3.921	Claire	0.444	<0.001	20.7

For Review Only

	WC.0213051	4D	56.9	17	28.97	yes	1.16	4.647	Robigus	0.471	<0.001	27.8	5.376	Robigus	0.471	<0.001	38.9
Grain yield	WC.0227571	7B	16.5	328	5.62	yes	0.66	0.354	Robigus	0.070	<0.001	13.9	0.235	Robigus	0.070	0.001	6.2

For Review Only

Supplementary table 4. Sources of variation related to asparagine and falling number screened in this study in the Claire x Robigus mapping population. Chr. (chromosome).

Source of variation	Claire	Robigus	Chr.	Reference
ASN-B2 PAV	Absent	Absent	3B	Oddy et al., 2021
ASN-B1	Non-functional	Functional	5B	Oddy et al., 2021
ASN-A3.1	Non-functional	Non-functional	1A	Oddy et al., 2021
Rht-B1	*Rht-B1a* (WT)	*Rht-B1b* (Dwarf)	4B	Wilkinson et al., 2020
Rht-D1	*Rht-D1b* (Dwarf)	*Rht-D1a* (WT)	4D	Wilkinson et al., 2020
T. dicoccoides introgression	Absent	Present	4A	Przewieslik-Allen et al., 2021
TaMKK3A	A	A	4A	Shorinola et al., 2016
PM19-A1 promoter InDel	Deletion	Deletion	4A	Shorinola et al., 2016

For Review Only

Supplementary table 5. Physical locations of the Hagberg Falling Number (HFN), asparagine (Asn), and lysine (Lys) QTL in Chinese Spring. Chr. (chromosome), Mbp (megabase pair location), CI (confidence interval), bp (base pair).

Trait	Chr.	Peak (Mbp)	Lower CI (Mbp)	Upper CI (Mbp)	QTL size (bp)	No. of genes
HFN	4A	733	691	745	54,058,906	824
Asn	4B	601	533	632	96,765,195	754
Lys	1A	593	590	594	4,471,109	50

For Review Only

For Review Only

Supplementary table 6. Physical location of the lysine QTL in the wheat pangenome (chromosome level assemblies) and gene content.

Genome	Chr	Lower CI	Upper CI	QTL size	No. of genes
IWGSC	1A	590	594	4,471,109	50
ArinaLrFor	1A	598	603	4,559,393	55
Jagger	1A	592	596	4,481,576	60
Julius	NA	NA	NA	NA	NA
Lancer	1A	591	595	4,548,456	59
Landmark	1A	593	595	2,101,319	38
Mace	1A	586	591	4,477,846	55
SY Mattis	1A	596	601	4,560,453	66
Norin61	1A	589	594	4,645,175	58
Stanley	scaffold_v3_2071	5	NA	NA	NA
